# Identification of Immune Related LRR-Containing Genes in Maize (*Zea mays* L.) by Genome-Wide Sequence Analysis

**DOI:** 10.1155/2015/231358

**Published:** 2015-10-22

**Authors:** Wei Song, Baoqiang Wang, Xinghua Li, Jianfen Wei, Ling Chen, Dongmin Zhang, Wenying Zhang, Ronggai Li

**Affiliations:** Key Laboratory of Crop Genetics and Breeding of Hebei Province, Institute of Cereal and Oil Crops, Hebei Academy of Agriculture and Forestry Sciences, Shijiazhuang 050035, China

## Abstract

A large number of immune receptors consist of nucleotide binding site-leucine rich repeat (NBS-LRR) proteins and leucine rich repeat-receptor-like kinases (LRR-RLK) that play a crucial role in plant disease resistance. Although many NBS-LRR genes have been previously identified in *Zea mays*, there are no reports on identifying NBS-LRR genes encoded in the N-terminal Toll/interleukin-1 receptor (TIR) motif and identifying genome-wide LRR-RLK genes. In the present study, 151 NBS-LRR genes and 226 LRR-RLK genes were identified after performing bioinformatics analysis of the entire maize genome. Of these identified genes, 64 NBS-LRR genes and four TIR-NBS-LRR genes were identified for the first time. The NBS-LRR genes are unevenly distributed on each chromosome with gene clusters located at the distal end of each chromosome, while LRR-RLK genes have a random chromosomal distribution with more paired genes. Additionally, six LRR-RLK/RLPs including FLS2, PSY1R, PSKR1, BIR1, SERK3, and Cf5 were characterized in *Zea mays* for the first time. Their predicted amino acid sequences have similar protein structures with their respective homologues in other plants, indicating that these maize LRR-RLK/RLPs have the same functions as their homologues act as immune receptors. The identified gene sequences would assist in the study of their functions in maize.

## 1. Introduction

Pathogens, including bacteria, fungi, oomycetes, and viruses, are a major threat to the global human food supply. They attack plants in an attempt to gain nutrients from them. During the course of evolution, both plants and pathogens have evolved methods to combat each other. Plants, like animals, are equipped with immune receptors for recognizing invading pathogens and activating innate immune responses [1, 2]. It is widely accepted that plant immune responses consist of two branches of resistance. The first branch involves plasma-membrane localized pattern recognition receptors (PRRs) that recognize the conserved microbial molecules referred to as pathogen-associated molecular patterns (PAMPs) [2, 3]. The second requires intracellular receptors that are proteins encoded by classical plant disease resistance (R) genes to detect the presence of pathogen proteins inside the host cell [4]. The largest group of plant immune receptors that were identified 20 years ago are cytoplasmic nucleotide binding site (NBS) leucine rich repeat (LRR) proteins encoded by R genes [5]. The NBS-LRR genes have been further subdivided into two main groups, based on their N-terminal structures [6–9]. The first group possesses a domain with homology to the intracellular signalling domains of the* Drosophila Toll* and mammalian interleukin-1 receptors and is referred to as TIR-NBS-LRRs or TNLs. The second, non-TNL, group is collectively known as CC-NBS-LRRs or CNLs, based on the presence of a predicted N-terminal coiled-coil domain in some, but not all, members of this group [10]. Studies on the structure of cloned R genes revealed that not all R genes encode NBS-LRR proteins. Several R genes encode transmembrane receptor-like kinase/proteins (RLK/RLP) that are one of the most important groups of cell surface receptors [4, 11]. A typical RLK contains an extracellular receptor domain (LRR) to perceive a specific signal, a transmembrane domain (TM) to anchor the protein within the membrane, and an intracellular cytoplasmic kinase domain to transduce the signal through autophosphorylation followed by further phosphorylation of downstream components to regulate gene expression [12–14].

Since the discovery of the first plant RLK in 1990, a small number of RLKs have been functionally characterized, such as flagellin sensing 2 (FLS2), tyrosine-sulfated peptide receptors PSKR1 and PSY1R [15–17]. The extracellular LRR domains in these proteins that act as cell surface immune receptors or components of receptor complexes contribute to plant defense/pathogen-recognition. Further studies on the molecular structure and function of flagellin reveal that numerous signal perception and transduction systems are needed in plants to recognize all potential invaders [18]. Indeed, more than 400 genes encoding RLK sequences with various receptor configurations are present in the genomic sequence of the* Arabidopsis thaliana*, of which 216 members contain an LRR in the extracellular domain [19]. In addition to RLKs, 149 R genes encoding NBS-LRR are also present in the* Arabidopsis thaliana* genome [20]. Therefore, the LRR-containing receptors play a crucial role in intercellular communication and disease resistance in plant immunity.

Since 1992 the first plant R gene,* Hm1*, which confers specific resistance against a leaf blight and ear mold disease of corn, was cloned. More than 100 R genes have been cloned from different plant species, of which approximately 80% encode R proteins with NBS-LRR domains [21]. To date, along with the development of bioinformatics technology and the availability of whole-genome sequences of several plants, such as* Arabidopsis*, rice,* Brachypodium distachyon*, maize, and sorghum, numerous NBS-LRR genes have been revealed [10, 20, 22–24]. However, reports on how many RLK/RLP genes are present in different plant species are rare. To further understand the importance of R genes, this study aims at identifying the sequences of R genes containing NBS-LRR and RLKs from the sequenced maize genome. In doing so, the R genes may be made available for breeding purposes. The bioinformatics analysis of the R gene homologues provides a definitive resource for the ongoing functional and evolutionary studies of this large plant gene family. Meanwhile, maize (*Zea mays *L.) is a food staple in many regions of the world and is used for animal feed and ethanol fuel. It is the world's most extensively grown crop and has the highest world-wide production in all cereal crops [http://faostat.fao.org/]. In addition to its economic value, maize is also an important model plant for studies in plant genetics, physiology, and development. Thus, our work will shed more light on the maize immune system and the findings will provide a strong groundwork for the isolation of candidate R genes in maize.

## 2. Methods

### 2.1. The Maize Genome

The complete genome sequence (RefGen_v3) of* Zea mays* (maize inbred line B73) collected in Ensemble (http://plants.ensembl.org/Zea_mays/Info/Index) was used in the genomic analysis of encoding LRR-receptor-like genes.

### 2.2. Identification of NBS-LRR and LRR-RLK Encoding Genes

Plant Gene Family Database (PGF-DB), a database for analysis of gene families from* Oryza sativa*,* Sorghum bicolor*,* Zea mays*, and* Arabidopsis thaliana*, contains more than ten thousands gene families constructed by the Markov clustering (MCL) method and the complete linkage method of BLASTP. By using keyword “NB-LRR” to search for gene family in PGF-DB (http://rapdb.dna.affrc.go.jp/) we identified one relevant gene family, “NB-ARC.” This family includes a number of maize genes that are homologues of known rice NBS-LRR genes. The obtained maize genes were used as a set of candidate NBS-LRR genes to search the maize Ensemble database. First, the complete set of NBS-encoding genes was identified in the genome of* Zea mays* by reiterative process of using the NBS domain from the Pfam database (PF00931; http://pfam.sanger.ac.uk/search). The threshold expectation value (*E*-value) was set to 10^−4^ which corresponds to that described in rice and* Arabidopsis* [20, 22]. In the second step, a set of LRR-encoding genes was identified using the conserved LRR-domain from the Pfam database (PF00560, PF08263, and PF12799) and then the encoding NBS-LRR genes were identified from the above two sets of NBS- and LRR-encoding genes. Sequences found multiple times were identified by multiple sequence alignments using Clustal W2 [25] and the redundant sequences were manually removed. All of the corresponding NBS-LRR candidate proteins were surveyed to determine whether they encoded TIR or CC motif. This survey was based on the Pfam database (http://pfam.xfam.org/search) and used SMART (Simple Modular Architecture Research Tool: http://smart.embl-heidelberg.de/) protein motif analysis and COILS, a program for detecting coiled coil (CC) domains (http://www.ch.embnet.org/software/COILS_form.html). The NBS-LRR candidate protein was filtered out in sequences lacking the TIR or CC motif.

Using a similar method to retrieving NBS-encoding genes as described previously, a complete set of RLK-encoding genes were retrieved from the maize Ensemble database by using the protein kinase catalytic domain (PF00069) from the Pfam database. The LRR-RLK genes were then identified using the conserved LRR-domain (PF00560, PF08263, and PF12799) from the identified RLK-encoding genes.

### 2.3. Gene Locations and Definitions of Gene Cluster and Gene Duplication

The positions of all the NBS-LRR and LRR-RLK genes on maize chromosomes were defined by Ensemble Genomes Search (http://plants.ensembl.org/Zea_mays/Info/Index) using the gene names. If two or more NBS-LRR/LRR-RLK genes resided within 200 kb, a gene cluster was defined based on Houb's definition of a gene cluster [26]. If two of the paired genes were located in a cluster, this pair was designated as paired genes; if three or more genes were located within 200 kb, these genes were designated as multigenes. The gene-duplication events of NBS-LRR and LRR-RLK genes were also investigated in accordance with the below criteria when the encoded amino acid sequence was used as a query in BLASTP searches for possible homologues in the* Zea mays* genome: (1) the sequence alignment covered >70% of the longer gene; (2) the aligned region had an 70% identity.

### 2.4. Alignment and Analysis of Sequence

Proteins encoded by LRR-RLK genes are crucial cell surface immune receptors for plant disease resistance. The LRR-RLK homologues in maize were found by searching the available maize nonredundant protein sequences database (taxid:4577) with BLASTP (http://blast.ncbi.nlm.nih.gov/Blast.cgi). The scoring parameters for BLASTP search were set as default: BLOSUM62 was used in the protein weight matrix, gap costs were set as 11 for existence and 1 for extension, and compositional adjustment was set as conditional compositional score matrix adjustment. The sequence with the highest score (the lowest* E* value and the highest identity) was categorized with LRR-RLK from other plants that had the same homology and phylogenetic grouping. Homology analysis of the maize LRR-RLK protein sequences with their homologue sequences was performed using MatGat software v2.02 [27]. The protein signature was also analyzed using the simple modular architecture research tool (SMART) [28]. Multiple sequence alignments were generated using Clustal W2 [25]. Based on a ClustalW multiple alignment phylogenetic tree, a similar one was constructed using the neighbor-joining method within the MEGA5 program [29] and bootstrapped 1,000 times.

## 3. Results 

### 3.1. Identification and Classification of NBS-LRR and LRR-RLK Encoding Genes

Availability of the complete* Zea mays* genome sequence (maize inbred line B73) has made it possible for the first time to identify all the LRR-containing receptor-like genes in this plant species [24]. From the first step of NBS-filter, a total of 217 NBS-encoding genes were identified in the genomic sequence of maize inbred line B73 and collected in Ensemble. In the second step of LRR-filter, 62 of the NBS-encoding genes were subsequently found not to be LRR-encoding genes. Of the remaining genes, 151 were identified to have an NBS-LRR structure and were surveyed using Pfam, SMART, and COILS to determine whether they encoded TIR, CC motifs. 147 genes were identified to contain a CC motif, but only four genes belong to the TNLs group (see Supplementary Table S1 in Supplementary Material available online at http://dx.doi.org/10.1155/2015/231358). The fact that most of these genes belong to CNLs in maize genome further suggests that the monocots are likely to lack the TIR genes [10, 22, 23, 30]. 64 out of 151 NBS-LRR genes were identified for the first time in this study [31] (marked in asterisk in Supplementary Table S1). The majority of these newly found genes are located on chromosomes 2, 4, and 7 (11, 18, 7, resp.). Upon initial searching of the LRR-RLK genes, 1521 protein-kinase-encoding genes were identified in the maize genome. Of these genes, 226 genes were found to be LRR-RLK protein encoding genes in a following reiterative process (Supplementary Table S2).

In maize whole-genome sequences, at least 39,469 protein-coding genes have been identified. NBS-LRR genes accounted for approximately 0.38% of all the protein-coding genes in this species. The relative proportion of these genes was over three times lower than that in the rice genome (1.23%) but slightly higher than that in the sorghum genome (0.18%) [22–24, 32, 33]. Additionally, the LRR-RLK encoding genes accounted for approximately 0.57% of maize protein-coding genes, 1.5 times higher than NBS-LRR genes. Furthermore, comparing with LRR-RLK gene numbers identified in maize, sorghum (*Sorghum bicolor*) had similar results with 208 [34] and 211 [35] in different studies. However the numbers in rice (*Oryza sativa*) varied widely from different studies which are 177 [34], 309 [36], and 353 [35], respectively.

### 3.2. Chromosomal Distribution of Maize NBS-LRR and NBS-RLK Encoding Genes

**Figure 1:**
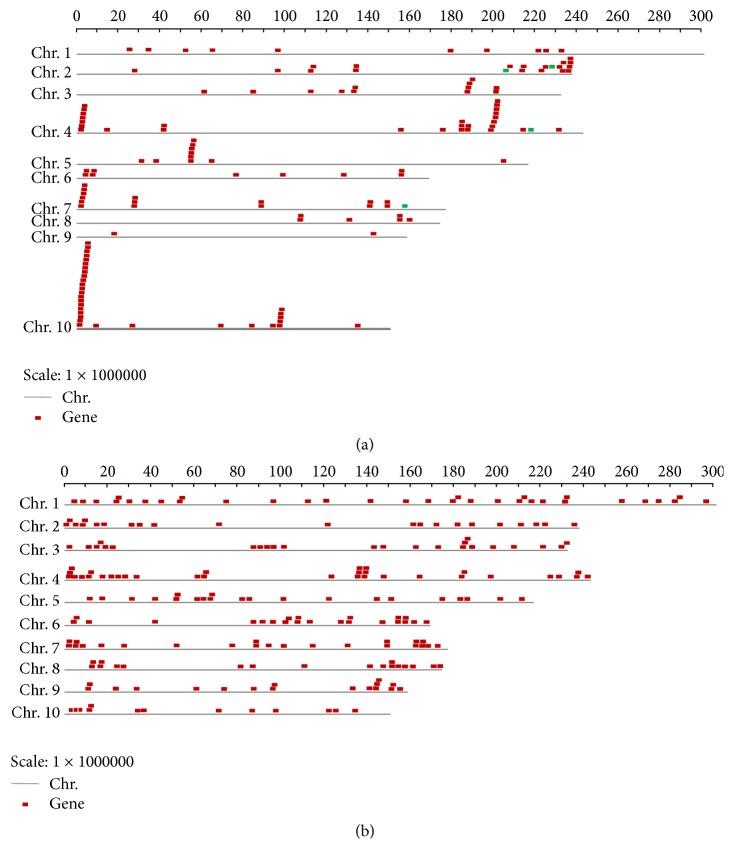
Physical locations of the* Zea mays R* genes encoding LRR: (a) physical locations of NBS-LRR genes; (b) physical locations of LRR-RLK genes. The boxes above the chromosomes (Chr.; gray bars) indicate the approximate locations of each gene. Red boxes indicate the CC-NBS-LRR genes, while green boxes indicate TIR-NBS-LRR genes. Chromosome lengths are shown in megabase pairs on the scale at top.

NBS-LRR type R genes were identified on each of ten maize chromosomes. The genes are either located separately on each individual chromosome or in gene clusters on a single chromosome (see Section 2 for detailed definition). Their distribution on chromosomes is nonrandom and uneven. For example, chromosome 9 contains only two NBS-LRR genes, while chromosomes 10, 4, and 2 contain 32, 28, and 18 NBS-LRR genes, respectively, with about 21.19% of those genes locating on chromosome 10 (Figure 1(a) and Table 1). Notably, two larger clusters have been found on chromosome 10, with one containing 14 genes and the other containing 5 genes (Figure 1(a) and Table 2). Similar to the distribution of sorghum NBS-LRR gene clusters [23], most of the maize NBS-LRR gene clusters were located at the distal end of each chromosome (Figure 1).

LRR-RLK genes are also located on each individual maize chromosome. However, their distribution on chromosomes is relatively even, unlike the clustered NBS-LRR genes (Figure 1(b) and Table 2). The number of LRR-RLK genes on each chromosome is between 13 (on Chr. 10) and 32 (on Chr. 4).

### 3.3. Duplications of NBS-LRR and LRR-RLK Genes

During evolution, both segmental duplication and tandem duplication have contributed to the large number of gene families in plants [37]. The gene duplications have greatly expanded the NBS gene family in both monocot and dicot lineages [20, 22, 23]. In this study, the duplication of NBS-LRR and LRR-RLK genes was confirmed by a BLASTP comparison of all the predicted maize proteins against each other. A total of 56 out of the 151 NBS-LRR genes were duplicated and they were subsequently divided into 16 multigene families (Tables 2 and 3). The maximum number of family members was 14, which cluster at the distal end of chromosome 10 (Figure 1), and the average number of family members was 3.5.

In contrast to NBS-LRR genes, 46 multigene families that contain 110 out of 226 LRR-RLK genes were identified in the maize genome (Tables 2 and 4). Among these multigene families, the percentage in LRR-RLK genes (48.67%) was higher than that in NBS-LRR genes (37.09%), demonstrating that almost half of the LRR-RLK genes were duplicated. Furthermore, the maximal number of family members in LRR-RLK genes was lower than that in NBS-LRR genes (5 and 14, resp.; Table 2). The average number of LRR-RLK members per multigene family was 2.4 and also lower than that of NBS-LRR members per multigene family. This result revealed that LRR-RLK genes are highly diverse NBS-LRR genes within the maize genome. More interestingly, no pairs of NBS-LRR genes were found on duplicated chromosomal segments, while 36 pairs of LRR-RLK genes were found. This indicates that the expansion of LRR-RLK genes may have been created via duplication and, subsequently, diversifying selection.

### 3.4. Molecular Characterization and Phylogenetic Analysis of Maize LRR-RLKs

**Figure 2:**
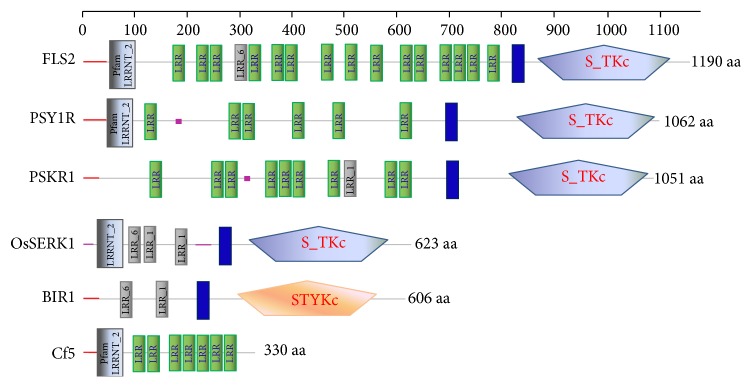
Schematic diagram of the protein features of the identified LRR-RLKs. This diagram was constructed using the predicted amino acid (aa) sequences from retrieved proteins and SMART online program. The size of each protein is presented with a grey line. The predicted domains are highlighted in each protein. The signal peptides are shown as red boxes, while the low complexity regions and transmembrane regions are shown as pink boxes and blue boxes, respectively. LRRNT_2: leucine rich repeats domains that consist of 2–45 motifs of 20–30 amino acids in length that generally fold into a horseshoe shape. LRR: leucine rich repeat domain; S_TKc: serine/threonine protein kinases, catalytic domain; STYKc: dual-specificity serine/threonine/tyrosine kinase domain.

To identify the homologues of the LRR-RLKs in maize, we used known plant LRR-RLK protein sequences to search the maize protein database. Out of the 226 identified LRR-RLK genes, six homologues were found, including FLS2 (GRMZM2G080041), Cf5 (GRMZM2G107872), PSY1R (GRMZM2G177570), PSKR1 (GRMZM2G080537), BIR1 (GRMZM2G121565), and OsSERK1 (GRMZM2G150024). All the predicted amino acid sequences of these genes contain a signal peptide, extracellular LRR domains, a single-pass transmembrane domain (TM), and an intracellular kinase domain except for Cf5 (Figure 2). Maize FLS2, OsSERK1, PSY1R, and PSKR1 are typically LRR-serine/threonine protein kinases, while BIR1 is dual-specificity serine/threonine/tyrosine kinase but Cf5 is a transmembrane LRR-receptor-like protein. At the amino acid level, the identified maize LRR-RLK/RLPs shared the highest identity (or similarity) within family members (Table 5). Meanwhile, maize LRR-RLK/RLP proteins share a higher identity (or similarity) to their monocots homologues than dicots homologues.

**Figure 3:**
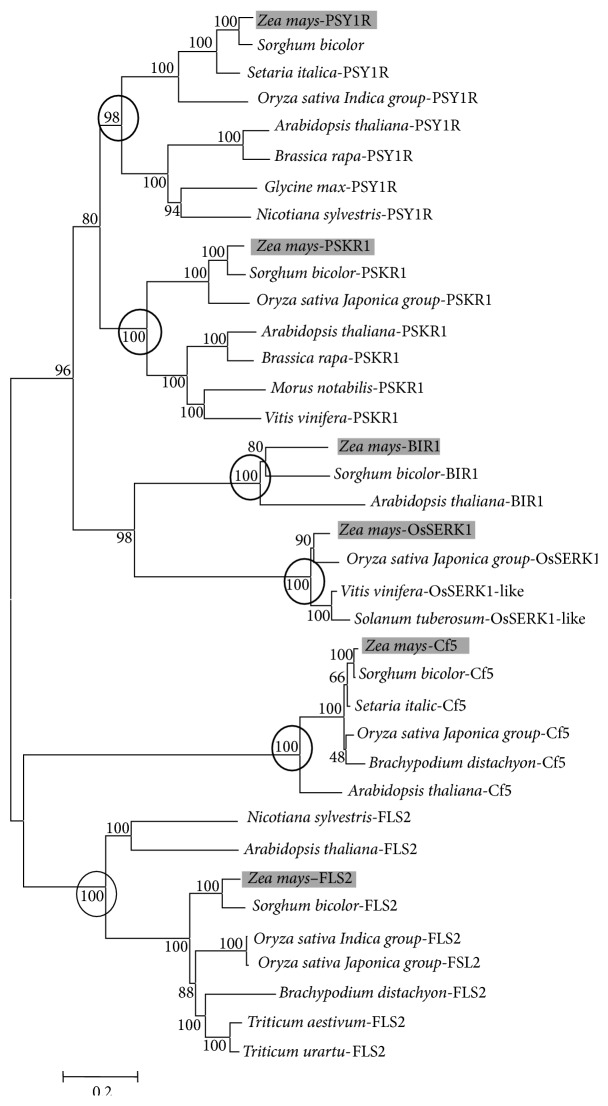
Unrooted phylogenetic tree of the seven identified LRR-RLKs showing the relationship with their homologues in other plants. This tree was constructed from a ClustalW generated multiple sequence alignment of amino acid sequences using the neighbor-joining method in MEGA5. Node values indicate percentage bootstrap values derived from 10,000 replications. The maize LRR-RLKs reported in this study are shaded in grey. The accession numbers of the sequences used for the phylogenetic analysis are as follows:* Zea mays* PSY1R [XP 008644981], PSKR1 [XP 008645956], BIR1 [NP 001147794], OsSERK1 [XP 008678722], Cf5 [NP 001132758], and FLS2 [XP 008668880];* Sorghum bicolor* PSY1R [XP 002437497], PSKR1 [XP 002454207], BIR1 [XP 002450551], Cf5 [XP 002465654], and FLS2 [Sb06g028760];* Setaria italica* PSY1R [XP 004966176] and Cf5 [XP 004985227];* Oryza sativa Indica Group* PSY1R [AAU12600] and FLS2 [CAH68341];* Oryza sativa Japonica Group* PSKR1 [BAD23737], OsSERK1 [AAU88198], Cf5 [NP 001049352], and FSL2 [CAE02151];* Brachypodium distachyon* Cf5 [XP 003558509] and FLS2 [BRADI5G21960];* Triticum aestivum* FLS2 [6E8764762];* Triticum urartu* FLS2 [EMS63184];* Arabidopsis thaliana* PSY1R [NP 177374], PSKR1 [NP 178330], BIR1 [NP 568696], Cf5 [NP 200932], and FLS2 [NP 199445];* Brassica rapa* PSY1R [XP 009105890] and PSKR1 [XP 009129163];* Glycine max* PSY1R [XP 003520891];* Nicotiana sylvestris* PSY1R [XP 009759322] and FLS2 [XP 009801818];* Morus notabilis* PSKR1 [EXC37937];* Vitis vinifera* PSKR1 [XP 002273186] and OsSERK1 [XP 002270847];* Solanum tuberosum* OsSERK1 [NP 001275293].

In order to elucidate the relationships among the maize LRR-RLK/RLP genes with their homologues in other plants, the encoded amino acid sequences of LRR-RLK/RLP genes (see Supplementary Material 2) were used to construct a neighbor-joining phylogenetic tree with the multiple sequence alignments (Figure 3). The phylogenetic tree showed that all of the six maize LRR-RLK/RLP protein sequences were grouped with their respective homologues, confirming their phylogenetic relationships. The tree also indicated that there were two distinct clades: one clade includes PSY1R, PSKR1, BIR1, and OsSERK1 which are typical LRR-RLKs and the other includes FLS2 and Cf5, both known as PRRs in dicots as they contain a more similar LRR domain. This is supported by the fact that these maize LRR-RLK/RLPs are grouped with their homologues from other plant species. It can be concluded that the maize LRR-RLK/RLPs are more close to that from monocots than dicots members.

## 4. Discussions

A genomic analysis of the disease-resistance genes encoding NBS has been extensively investigated in many plant species. However no TIR-NBS-LRR genes were reported in maize and the LRR-RLK genes were rarely characterized, although the first plant RLK was identified in maize twenty years ago [38]. In the present study, we discovered for the first time four TIR-NBS-LRR genes out of 151 NBS-LRR genes and 226 LRR-RLK genes from the maize whole-genomic sequence database and further characterized six proteins encoded by LRR-RLK/RLP genes. The putative LRR domain in these proteins is likely to act as a ligand-binding domain to recognize pathogens as part of the maize immune system.

The maize genome sequence data employed in the present study is more accurate than in previous studies as gaps were also sequenced, which enabled us to estimate the actual number of NBS-LRR encoding genes. Thus, additional 64 NBS-LRR genes in maize were identified compared to 109 [31] or 95 [39] NBS-LRR genes in previous investigations. It is interesting that the newly found genes on chromosome 7 are in clusters or pairs, indicating that the local tandem duplications are possibly responsible for gene expansion. 226 LRR-RLK genes were discovered in maize in this study, which is in line with the number of* Sorghum bicolor* (211) and* Arabidopsis thaliana* (213), but much less than that of* Oryza sativa* (353) [35].

Previous studies have shown that rice diverged from the progenitors of maize and sorghum about 60 million years ago, whereas maize and sorghum evolved from a common ancestor about 25 million years ago [40]. The NBS-LRR genes are much less frequent in maize than in rice or sorghum, in which 480 and 211 NBS-LRR genes were identified, respectively [22, 23]. This suggests that loss of NBS-LRR genes in maize and sorghum was rapid after the split with rice especially in maize. The distribution of the number of NBS-LRR genes on each chromosome confirmed previous findings, although the total number found was different [31]. For example, chromosome 10 contains the greatest number of NBS-LRR genes, whereas chromosome 9 has the least. Additionally, NBS-LRR genes are most abundant on maize chromosome 10, and their homologues are located in chromosome 11 in rice and chromosome 8 in sorghum [33, 41]. Clustering of NBS-LRR genes in the genomic regions in various species suggests that there are chromosomal hot spots in which the NBS-LRR genes are duplicated. Additionally, 14 NBS-LRR genes are located in maize chromosome 10, suggesting that these genes originated by tandem duplications and subsequently evolved under selective pressure in this region. Maize NBS-LRR genes mostly encode the CC-type of N-terminal domains. Only four maize TIR-encoding genes (GRMZM2G319375, GRMZM2G402165, GRMZM2G132403, and GRMZM2G302279) were identified. Similar findings were also reported in other monocots, such as rice (only one TIR-encoding gene identified), sorghum (two TIR-encoding genes), and* Brachypodium distachyon* (none identified) [10, 22, 33]. In contrast, the TIR encoding genes have extensively expanded in dicots, with 98 found in Arabidopsis and 78 in poplar [20, 30]. The CC-type NBS-LRR genes from dicots and monocots tend to cluster together on chromosomes suggesting that the CC-type NBS-LRR genes originated before the divergence of the monocots and dicots [20, 22, 23]. Additionally, TIR-type genes are likely to have been lost from the grass species rather than having arisen from plant evolution after the monocot/dicot separation as there are less TIR-type genes in monocots than dicots, although the reason remains unclear [42, 43].

In maize, 27.8% of the NBS-LRR genes are located in gene clusters, a much lower proportion than in sorghum at 97% [23]. It has also been reported that the highest proportion of clustered NBS-LRR genes has been found in Arabidopsis and rice [20, 22]. Moreover, there is potentially less duplication and fewer multigene families in maize than sorghum and rice. This peculiar distribution in maize might be caused by more dispersed-recombination than duplication as the maize genome is approximately 3 times bigger than the sorghum genome [33]. In contrast, the LRR-RLK genes are not distributed in clusters but are scattered throughout the maize genome. Nearly 50% of LRR-RLK genes have been duplicated, indicating that a whole-genome wide duplication event could result in the expansion of LRR-RLK genes in maize. The difference genome location and copy number between NBS-LRR genes and LRR-RLK genes suggest that they may have evolved from two different ancestors and have different roles in acting as receptors in plant immune systems. Additionally, the genome-wide distribution of maize NBS-LRR and LRR-RLK genes indicates that a large number of different loci are related to the immune system and that the maize resistance system is very complex.

The number of LRR-RLK genes found in Arabidopsis was similar to that in maize. More than 400 genes were identified with RLK configurations that can be classified into at least 21 structural classes based on their extracellular domains, in which the LRR-RLKs represented the largest group consisting of 216 members [19]. The presence of LRR-RLK genes in both dicot and monocot suggests that the size of this gene family may have been similar to the present-day level before the diversification of the land plant lineages.

The plant RLKs have been functionally characterised in some studies and are implicated to be involved in a diverse range of signalling processes, including brassinosteroid signalling via brassinosteroid insensitive 1 (BRI1) [44], recognition of flagellin by FLS2 [15], and bacterial resistance mediated by Xa21 [11]. So far, none of 226 LRR-RLKs in maize have been functionally studied. The LRR-RLK genes identified in this paper will be invaluable for gene function analysis. In addition, six maize LRR-RLK/RLPs were found to contain an extracellular ligand-binding domain (LRR domain), a single membrane-spanning domain, and a C-terminal intracellular protein kinase domain (tyrosine or serine/threonine rich region) and thus it is likely that they have the same function as their homologues in other plant species. For example, FLS2 functions as an immune receptor sensing the bacterial flagellin in maize as it does in Arabidopsis. The extracellular LRR domain to recognize the peptide fig22 [45], maize BIR1, as a BAK1- (BRI1-associated receptor kinase 1-) interacting receptor-like kinase, works together with BAK1 to negatively regulate cell death and defense responses [12, 46, 47]. Other than OsSERK1 (GRMZM2G150024 on Chr. 4), two more SERK (Somatic Embryogenesis Receptor-Like Kinase) family members, named ZmSERK1 (GRMZM5G870959 on Chr. 10) and ZmSERK2 (GRMZM2G115420 on Chr. 5), had been characterised in maize previously [48]. Besides the role in somatic embryogenesis, the SERK family members have been associated with R-gene resistance, such as Mi-1 against potato aphids in tomato plants [49] and Xa21 against* Xanthomonas oryzae *pv.* oryzae* (Xoo) in rice [50]. Although three members of the SERK family have been found in maize, whether they have the same functions in disease resistance as seen in tomato and rice remains to be determined.

In conclusion, this study has identified a number of the maize LRR-receptor-like genes and characterised a number of LRR-RLK/RLP genes based on their structural domains, physical chromosomal locations, and phylogenetic relationships. These sequences will aid in the study of their functions in maize.

## Supplementary Material

Short description for the Supplementary Material 1: One hundred and fifty one NBS-LRR genes and 226 LRR-RLK genes were identified which are listed in the Supplementary Material 1. 64 out of the 151 NBS-LRR genes were identified for the first time, which are marked with asterisks in Supplementary Table S1.
Short description for the Supplementary Material 2: In order to elucidate relationships among the maize LRR-RLK/RLP genes with their homologues in other plants, the encoded amino acid sequences of LRR-RLK/RLP genes were used to construct a neighbour-joining phylogenetic tree with the multiple sequence alignments. The sequences used for phylogenetic analysis are listed in Supplementary Material 2.

## Figures and Tables

**Table 1:** The distribution of LRR-receptor genes on maize chromosome.

Chromosome	NBS-LRR gene	LRR-RLK gene
1	11	31
2	22	22
3	12	24
4	28	32
5	10	21
6	10	22
7	18	23
8	6	18
9	2	20
10	32	13
Total	151	226

**Table 2:** Comparison of duplications of genes of NB-LRR and LRR-RLK in maize genome.

Gene type	NBS-LRR	LRR-RLK
Total number of genes	151	226

Single-gene families	94	117
Single-genes	94	117
Singletons	56	100

Multigene families	16	46
Multigenes	56	110
Maximal family members	14	5
Average members per family	3.5	2.4
Paired genes	14	36
Number of gene clusters	9	0
Clustered genes	42	0
Percentage of multigenes	37.09	48.67

**Table 3:** The duplicated NBS-LRR genes in maize genome.

Number	Duplicated gene	Chr. location
1	GRMZM5G896901	1: 65769722–65771244
	GRMZM2G454718	5: 55977976–55979514

2	GRMZM2G443525	1: 164103203–164108542
	GRMZM2G044724	3: 85672960–85676903

3	GRMZM2G076474	2: 113990307–113993559
	GRMZM2G142680	10: 94979016–94982685

4	GRMZM2G065692	2: 134668301–134679003
	LOC100383650	2: 134763564–134769552
	GRMZM2G003755	2: 134763419–134769548

5	GRMZM2G074496	2: 225896403–225899247
	AC195587.4_FGP004	6: 78774141–78783661

6	GRMZM2G070503	2: 236854869–236856413
	GRMZM2G026083	7: 2515278–2517156
	GRMZM2G026189	7: 2525679–2527394
	LOC103631898	7: 2539874–2544540
	GRMZM2G403407	7: 2563208–2564699
	LOC103631902	7: 2569872–2585021
	GRMZM2G060714	7: 28599482–28602195
	GRMZM5G827455	7: 2582504–2583985

7	GRMZM2G094664	2: 237541842–237545769
	LOC103648313	2: 237541778–237547469

8	GRMZM2G064015	3: 132410799–132412650
	GRMZM2G302279	3: 132637536–132639351

9	GRMZM2G005347	4: 203407679–203411878
	GRMZM2G005452	4: 203456994–203463296
	GRMZM2G167049	4: 203818715–203824772

10	GRMZM2G162098	5: 55459467–55463724
	GRMZM2G091672	5: 55578326–55582323
	GRMZM2G091696	5: 55590775–55599910
	GRMZM2G105428	5: 55809843–55813411

11	GRMZM2G306727	6: 7232528–7235241
	GRMZM2G334584	6: 8100151–8105044

12	LOC103630086	6: 128972997–128982259
	GRMZM2G397785	10: 97377587–97383756

13	GRMZM2G169584	8: 155079613–155081884
	GRMZM2G169571	8: 155119523–155121206

14	GRMZM2G180244	10: 2052395–2058076
	GRMZM2G180254	10: 2127268–2133476
	GRMZM2G004412	10: 2987324–2996389
	GRMZM5G819919	10: 2842742–2846127

15	AC152495.1_FGP002	10: 3283523–3287419
	AC152495.1_FGP003	10: 3299975–3307226
	AC152495.1_FGP010	10: 3372154–3376038
	AC152495.1_FGP015	10: 3404105–3405367
	AC152495.1_FGP017	10: 3441445–3444666
	GRMZM5G879178	10: 3445065–3449748
	GRMZM2G069382	10: 3571990–3590706
	GRMZM2G083246	10: 3645995–3648135
	GRMZM2G143769	10: 3687121–3688770
	GRMZM2G443939	10: 3700999–3705985
	GRMZM2G349565	10: 3767328–3769922
	GRMZM2G003625	10: 3849433–3854134
	GRMZM2G061742	10: 3907357–3911655
	GRMZM2G005134	10: 3983698–3989918

16	GRMZM2G319375	2: 206507648–206508569
	GRMZM2G394261	7: 158297463–158301857

**Table 4:** The duplicated LRR-RLK genes in maize genome.

Number	Duplicated gene	Chr. location
1	GRMZM2G371137	1: 5803454–5808250
	GRMZM2G172014	9: 153777111–153781389

2	GRMZM2G009818	1: 9800492–9804655
	GRMZM2G438840	9: 152523091–152526601
	GRMZM2G168603	5: 68680529–68684140

3	GRMZM2G153393	1: 17640006–17655596
	GRMZM2G330907	9: 150263840–150275563
	GRMZM2G004572	1: 141080538–141086039

4	GRMZM2G038165	1: 26120398–26128604
	GRMZM2G069201	3: 185481180–185489362
	GRMZM2G089461	6: 154565943–154575228
	GRMZM2G174585	6: 154839078–154847341

5	GRMZM2G043584	1: 30258404–30262357
	GRMZM2G141517	7: 4082077–4086056
	GRMZM2G072569	1: 282595023–282599365

6	GRMZM5G886952	1: 38791249–38799875
	GRMZM2G479243	9: 140610164–140617939

7	GRMZM2G084587	1: 76878185–76889886
	GRMZM2G122717	4: 27843728–27845162

8	GRMZM2G093809	1: 180006011–180009342
	GRMZM2G080503	3: 92276172–92279783

9	GRMZM2G461278	1: 180366146–180370208
	GRMZM2G147857	3: 93326318–93329971

10	GRMZM2G428554	1: 210641732–210645615
	GRMZM2G011806	4: 61742558–61747904

11	GRMZM2G001812	1: 233516803–233520836
	GRMZM2G112309	5: 30237719–30241622

12	GRMZM2G137788	1: 268802186–268806287
	GRMZM2G158359	5: 10425114–10429156

13	GRMZM2G114276	2: 16005190–16009431
	GRMZM2G016477	10: 136139046–136143214

14	GRMZM2G021619	2: 36708164–36710640
	GRMZM2G167280	10: 126603529–126608907

15	GRMZM2G073928	2: 161907176–161910550
	GRMZM2G432642	7: 18436556–18440330

16	GRMZM2G163724	2: 164310419–164313687
	GRMZM2G072868	7: 50383762–50386524

17	GRMZM2G002569	2: 182995589–182999190
	GRMZM2G084248	7: 117574984–117578965

18	AC233861.1_FG001	2: 189738061–189741584
	GRMZM2G149051	7: 130969891–130973647
	GRMZM2G039431	4: 197994710–197998218
	GRMZM2G131609	6: 129696721–129700755
	GRMZM2G463493	8: 17060563–17064940

19	GRMZM2G172429	2: 201545881–201550118
	GRMZM2G313643	7: 150075579–150080212

20	GRMZM2G162781	2: 213192905–213197565
	GRMZM2G081857	7: 167142415–167147144

21	GRMZM2G169681	2: 219686483–219692413
	GRMZM2G068398	4: 10223589–10229102

22	GRMZM2G349875	2: 221419815–221427366
	GRMZM2G104384	4: 17311139–17323011

23	GRMZM2G012861	3: 2763089–2767869
	GRMZM2G127687	8: 26863212–26867537

24	GRMZM2G010693	3: 12429859–12434725
	GRMZM2G067675	8: 17304016–17309182

25	GRMZM2G463574	3: 16573593–16577181
	GRMZM2G421669	8: 14848451–14851761

26	GRMZM2G138338	3: 147442361–147446195
	GRMZM2G059117	8: 157002878–157006630

27	GRMZM2G447447	3: 161622655–161626810
	GRMZM2G306771	5: 123289050–123293128

28	GRMZM2G145753	3: 184727722–184731545
	GRMZM2G128315	6: 154358844–154362599

29	GRMZM2G465771	3: 185367968–185371929
	AC218972.3_FG004	8: 170198252–170201068
	AC214817.3_FG004	6: 154514150–154517066

30	GRMZM2G078926	3: 209624891–209634088
	GRMZM2G059497	8: 149851364–149863364

31	GRMZM2G339540	3: 224241290–224255475
	GRMZM2G050548	8: 141765271–141771284

32	GRMZM2G438007	4: 65769675–65773352
	GRMZM2G092604	7: 79119386–79123848

33	GRMZM2G150024	4: 124660672–124667062
	GRMZM2G115420	5: 176261637–176267226
	GRMZM2G384439	10: 71477986–71484046
	GRMZM5G870959	10: 121772419–121777860

34	GRMZM2G071396	4: 138248437–138252721
	GRMZM2G702599	4: 139022453–139031123
	GRMZM2G039665	4: 139156285–139159606

35	GRMZM2G150930	4: 165794972–165797318
	GRMZM2G171114	6: 103870389–103873917

36	GRMZM2G100234	4: 226544149–226547671
	GRMZM2G176206	5: 151922862–151926238

37	GRMZM2G451007	4: 237861330–237864849
	GRMZM2G177570	5: 51462007–51465569
	GRMZM2G474777	6: 88899974–88903683
	GRMZM2G104425	5: 81113414–81119465

38	GRMZM2G322348	4: 240249146–240253001
	GRMZM2G123314	8: 14843475–14846908

39	GRMZM2G126161	5: 42533252–42537728
	GRMZM2G100858	6: 91599104–91604620

40	GRMZM2G463904	5: 211800909–211813877
	GRMZM5G809695	6: 108369697–108377055
	GRMZM2G082855	9: 24074354–24081173

41	GRMZM2G125081	6: 5162305–5165886
	GRMZM2G045981	9: 146683625–146687407

42	GRMZM2G162531	6: 97773823–97791863
	GRMZM2G071573	9: 98256956–98262572

43	GRMZM2G449817	6: 104839663–104842894
	GRMZM2G350918	9: 74480747–74484663

44	GRMZM2G349665	6: 116081434–116084931
	GRMZM2G145720	9: 35179613–35185362
	GRMZM2G019317	5: 204644182–204648555

45	GRMZM2G149201	6: 130782221–130784726
	GRMZM2G151738	8: 89697764–89700024

46	GRMZM2G046729	9: 146340642–146344213
	AC233893.1_FG006	9: 146407378–146411588

47	GRMZM2G009770	9: 152025490–152029185
	GRMZM5G839644	10: 6940302–6943942

**Table 5:** Amino acid identities and similarities of LRR-RLK homologues from different plants^a^.

Gene	Organism	Identity (%)	Similarity (%)
FLS2	*Zea mays*	100.0	—
	*Sorghum bicolor*	87.6	92.4
	*Oryza sativa Indica*	68.8	80.9
	*Oryza sativa Japonica*	68.5	80.7
	*Triticum aestivum*	70.7	81.0
	*Triticum urartu*	46.0	53.4
	*Brachypodium distachyon*	56.1	67.3
	*Nicotiana sylvestris*	47.3	67.1
	*Arabidopsis thaliana*	44.4	66.0

Cf5	*Zea mays*	100.0	—
	*Sorghum bicolor*	98.2	98.8
	*Oryza sativa Japonica*	91.1	95.5
	*Setaria italica*	94.5	97.3
	*Brachypodium distachyon*	90.0	96.7
	*Arabidopsis thaliana*	73.6	83.9

OsSERK1	*Zea mays*	100.0	
	*Oryza sativa Indica*	87.0	91.2
	*Vitis vinifera*	83.9	88.9
	*Solanum tuberosum*	83.5	89.0

PSKR1	*Zea mays*	100.0	
	*Sorghum bicolor*	89.0	94.4
	*Oryza sativa Japonica*	77.5	87.5
	*Morus notabilis*	53.1	71.2
	*Brassica rapa*	47.8	65.4
	*Vitis vinifera*	53.0	71.3
	*Arabidopsis thaliana*	50.7	70.5

PSY1R	*Zea mays*	100.0	
	*Sorghum bicolor*	91.5	94.9
	*Setaria italica*	81.9	90.0
	*Oryza sativa Indica*	62.9	77.1
	*Brassica rapa*	46.3	64.3
	*Glycine max*	48.3	66.5
	*Nicotiana sylvestris*	47.2	66.1
	*Arabidopsis thaliana*	46.6	65.1

BIR1	*Zea mays*	100.0	
	*Sorghum bicolor*	65.5	82.3
	*Arabidopsis thaliana*	57.8	74.7

^a^The accession numbers of the sequences used for the homology analysis are listed in Figure 3.
