# Supplementary material for: Identification of Immune Related LRR-Containing Genes in Maize (Zea mays L.) by Genome-Wide Sequence Analysis
Source: Int J Genomics. 2015 Oct 22;2015:231358. doi: 10.1155/2015/231358 (PMC4645488; doi:10.1155/2015/231358)
Supplement: Supplementary file 1 — Short description for the Supplementary Material 1: One hundred and fifty one NBS-LRR genes and 226 LRR-RLK genes were identified which are listed in the Supplementary Material 1. 64 out of the 151 NBS-LRR genes were identified for the first time, which are marked with asterisks in Supplementary Table S1. Short description for the Supplementary Material 2: In order to elucidate relationships among the maize LRR-RLK/RLP genes with their homologues in other plants, the encoded amino acid sequences of LRR-RLK/RLP genes were used to construct a neighbour-joining phylogenetic tree with the multiple sequence alignments. The sequences used for phylogenetic analysis are listed in Supplementary Material 2. [file 231358.f1.docx]

**Supplementary Material 1.**

**Table S1. NBS-LRR encoding genes in maize genome.**

| **Gene** | **Protein Class** | **Chr. Location** | **Gene** | **Protein Class** | **Chr. Location** |
| --- | --- | --- | --- | --- | --- |
| GRMZM2G107872* | CC-NBS-LRR | 1:25423809-25428035 | GRMZM2G091672 | CC-NBS-LRR | 5:55578326-55582323 |
| GRMZM2G136513 | CC-NBS-LRR | 1:35137154-35141520 | GRMZM2G091696 | CC-NBS-LRR | 5:55590775-55599910 |
| GRMZM2G033519* | CC-NBS-LRR | 1:52527412-52539244 | GRMZM2G105428 | CC-NBS-LRR | 5:55809843-55813411 |
| GRMZM5G896901* | CC-NBS-LRR | 1:65769722-65771244 | GRMZM2G454718 | CC-NBS-LRR | 5:55977976-55979514 |
| GRMZM2G077937* | CC-NBS-LRR | 1:97510599-97513828 | GRMZM2G469414 | CC-NBS-LRR | 5:57234376-57240744 |
| GRMZM2G443525 | CC-NBS-LRR | 1:164103203-164108542 | GRMZM2G060583 | CC-NBS-LRR | 5:64802785-64807893 |
| GRMZM2G461269 | CC-NBS-LRR | 1:180359657-180365537 | GRMZM5G898898* | CC-NBS-LRR | 5:205233725-205237469 |
| GRMZM2G322748 | CC-NBS-LRR | 1:196725204-196728930 | GRMZM2G054946 | CC-NBS-LRR | 6:5444264-5447584 |
| GRMZM2G077068 | CC-NBS-LRR | 1:221288944-221294518 | AC193598.3_FG002 | CC-NBS-LRR | 6:5506218-5510111 |
| GRMZM2G045027 | CC-NBS-LRR | 1:225408071-225412051 | GRMZM2G306727* | CC-NBS-LRR | 6:7232528-7235241 |
| GRMZM2G150179 | CC-NBS-LRR | 1:232588727-232592939 | GRMZM2G334584 | CC-NBS-LRR | 6:8100151-8105044 |
| GRMZM2G032602 | CC-NBS-LRR | 2:29557868-29562262 | GRMZM2G082112* | CC-NBS-LRR | 6:98424341-98454553 |
| GRMZM2G450496 | CC-NBS-LRR | 2:97740216-97755349 | AC195587.4_FGP004 | CC-NBS-LRR | 6:78774141-78783661 |
| GRMZM2G076474 | CC-NBS-LRR | 2:113990307-113993559 | LOC103630086* | CC-NBS-LRR | 6:128972997-128982259 |
| GRMZM2G376708* | CC-NBS-LRR | 2:114028833-114057673 | GRMZM2G002656 | CC-NBS-LRR | 6:129006368-129016640 |
| GRMZM2G065692 | CC-NBS-LRR | 2:134668301-134679003 | GRMZM2G116335 | CC-NBS-LRR | 6:155791613-155793164 |
| LOC100383650* | CC-NBS-LRR | 2:134763564-134769552 | GRMZM2G440849* | CC-NBS-LRR | 6:155800098-155801930 |
| GRMZM2G003755 | CC-NBS-LRR | 2:134763419-134769548 | GRMZM2G337881* | CC-NBS-LRR | 7:2320875-2322495 |
| GRMZM2G319375* | TIR-NBS-LRR | 2:206507648-206508569 | GRMZM2G128693* | CC-NBS-LRR | 7:2362266-2363807 |
| GRMZM2G079082 | CC-NBS-LRR | 2:209789141-209793322 | GRMZM2G026083* | CC-NBS-LRR | 7:2515278-2517156 |
| GRMZM2G354013* | CC-NBS-LRR | 2:213970923-213974319 | GRMZM2G026189* | CC-NBS-LRR | 7:2525679-2527394 |
| GRMZM2G379770 | CC-NBS-LRR | 2:214870532-214873480 | GRMZM2G403407* | CC-NBS-LRR | 7:2563208-2564699 |
| GRMZM5G837251 | CC-NBS-LRR | 2:222145605-222148836 | LOC103631898* | CC-NBS-LRR | 7:2539874-2544540 |
| GRMZM2G074496 | CC-NBS-LRR | 2:225896403-225899247 | LOC103631902* | CC-NBS-LRR | 7:2569872-2585021 |
| GRMZM2G078013 | CC-NBS-LRR | 2:229691128-229697078 | GRMZM5G827455* | CC-NBS-LRR | 7:2582504-2583985 |
| GRMZM2G452954* | CC-NBS-LRR | 2:231089786-231091520 | GRMZM2G382273 | CC-NBS-LRR | 7:28278050-28283085 |
| GRMZM2G402165* | TIR-NBS-LRR | 2:232935649-232936614 | GRMZM2G038388 | CC-NBS-LRR | 7:28331519-28335396 |
| GRMZM2G444543 | CC-NBS-LRR | 2:233184126-233187916 | GRMZM2G060714* | CC-NBS-LRR | 7:28599482-28602195 |
| GRMZM2G070503* | CC-NBS-LRR | 2:236854869-236856413 | GRMZM2G163860* | CC-NBS-LRR | 7:89912626-89957628 |
| AC199068.2_FG017* | CC-NBS-LRR | 2:236154599-236156358 | GRMZM5G825192* | CC-NBS-LRR | 7:90816756-90817949 |
| LOC103648313* | CC-NBS-LRR | 2:237541778-237547469 | GRMZM5G827121* | CC-NBS-LRR | 7:140363758-140365602 |
| GRMZM2G094664 | CC-NBS-LRR | 2:237541842-237545769 | GRMZM2G176403* | CC-NBS-LRR | 7:140295380-140296477 |
| GRMZM2G396357 | CC-NBS-LRR | 2:237566351-237571362 | GRMZM2G397557 | CC-NBS-LRR | 7:148737081-148741183 |
| GRMZM2G091088 | CC-NBS-LRR | 3:61341905-61344669 | GRMZM2G028713 | CC-NBS-LRR | 7:148771846-148784786 |
| GRMZM2G044724 | CC-NBS-LRR | 3:85672960-85676903 | GRMZM2G394261* | TIR-NBS-LRR | 7:158297463-158301857 |
| AC230011.2_FGP002 | CC-NBS-LRR | 3:113913960-113920267 | GRMZM2G017603 | CC-NBS-LRR | 8:103901676-103904417 |
| GRMZM2G454039* | CC-NBS-LRR | 3:127874087-127877026 | GRMZM2G047152 | CC-NBS-LRR | 8:103983030-103986649 |
| GRMZM2G064015 | CC-NBS-LRR | 3:132410799-132412650 | GRMZM2G351921 | CC-NBS-LRR | 8:131064591-131072960 |
| GRMZM2G302279 | CC-NBS-LRR | 3:132637536-132639351 | GRMZM2G169584 | CC-NBS-LRR | 8:155079613-155081884 |
| GRMZM2G047652 | CC-NBS-LRR | 3:188433722-188435241 | GRMZM2G169571 | CC-NBS-LRR | 8:155119523-155121206 |
| GRMZM2G103135* | CC-NBS-LRR | 3:188513398-188515764 | GRMZM2G016802 | CC-NBS-LRR | 8:160073966-160077226 |
| GRMZM2G178704 | CC-NBS-LRR | 3:188572052-188573581 | GRMZM2G403151 | CC-NBS-LRR | 9:19238355-19245346 |
| GRMZM2G173647 | CC-NBS-LRR | 3:189832965-189836546 | GRMZM2G081458* | CC-NBS-LRR | 9:143592388-143593929 |
| AC213769.3_FG001* | CC-NBS-LRR | 3:202295637-202297205 | GRMZM2G180244 | CC-NBS-LRR | 10:2052395-2058076 |
| GRMZM2G116271 | CC-NBS-LRR | 3:202326228-202328969 | GRMZM2G180254 | CC-NBS-LRR | 10:2127268-2133476 |
| GRMZM2G308369* | CC-NBS-LRR | 4:1081917-1094326 | GRMZM2G437314 | CC-NBS-LRR | 10:2260118-2263935 |
| GRMZM2G455909 | CC-NBS-LRR | 4:1553768-1556576 | GRMZM5G819919* | CC-NBS-LRR | 10:2842742-2846127 |
| GRMZM2G455321* | CC-NBS-LRR | 4:1873582-1875342 | GRMZM2G004412 | CC-NBS-LRR | 10:2987324-2996389 |
| GRMZM2G311664* | CC-NBS-LRR | 4:1915881-1922172 | AC152495.1_FGP002 | CC-NBS-LRR | 10:3283523-3287419 |
| GRMZM2G013170* | CC-NBS-LRR | 4:1940774-1946421 | AC152495.1_FGP003 | CC-NBS-LRR | 10:3299975-3307226 |
| GRMZM2G171045* | CC-NBS-LRR | 4:2379673-2400194 | AC152495.1_FGP010 | CC-NBS-LRR | 10:3372154-3376038 |
| GRMZM2G448282* | CC-NBS-LRR | 4:2412312-2414762 | AC152495.1_FGP015 | CC-NBS-LRR | 10:3404105-3405367 |
| GRMZM2G304049 | CC-NBS-LRR | 4:42287260-42289282 | AC152495.1_FGP017 | CC-NBS-LRR | 10:3441445-3444666 |
| GRMZM2G025903* | CC-NBS-LRR | 4:42306205-42307763 | GRMZM5G879178* | CC-NBS-LRR | 10:3445065-3449748 |
| GRMZM2G333659 | CC-NBS-LRR | 4:15625718-15635892 | GRMZM2G069382 | CC-NBS-LRR | 10:3571990-3590706 |
| GRMZM2G059339* | CC-NBS-LRR | 4:157902482-157903591 | GRMZM2G083246* | CC-NBS-LRR | 10:3645995-3648135 |
| GRMZM2G521481* | CC-NBS-LRR | 4:176373677-176376135 | GRMZM2G083258 | CC-NBS-LRR | 10:3649253-3650329 |
| GRMZM2G380784* | CC-NBS-LRR | 4:185642204-185660442 | GRMZM2G143769 | CC-NBS-LRR | 10:3687121-3688770 |
| GRMZM2G158316* | CC-NBS-LRR | 4:185660976-185663417 | GRMZM2G443939 | CC-NBS-LRR | 10:3700999-3705985 |
| GRMZM2G111928* | CC-NBS-LRR | 4:185739331-185740582 | GRMZM2G349565* | CC-NBS-LRR | 10:3767328-3769922 |
| GRMZM5G880361* | CC-NBS-LRR | 4:187636692-187641029 | GRMZM2G003625 | CC-NBS-LRR | 10:3849433-3854134 |
| GRMZM2G050959 | CC-NBS-LRR | 4:189470957-189474033 | GRMZM2G061742 | CC-NBS-LRR | 10:3907357-3911655 |
| GRMZM2G067555* | CC-NBS-LRR | 4:198293916-198298368 | GRMZM2G005134 | CC-NBS-LRR | 10:3983698-3989918 |
| GRMZM2G327659 | CC-NBS-LRR | 4:200912556-200916516 | GRMZM2G350841 | CC-NBS-LRR | 10:5006086-5010500 |
| GRMZM2G039878* | CC-NBS-LRR | 4:201813148-201815687 | GRMZM2G381429 | CC-NBS-LRR | 10:9907952-9921133 |
| GRMZM2G005347 | CC-NBS-LRR | 4:203407679-203411878 | AC203972.3_FGP001 | CC-NBS-LRR | 10:27655922-27658633 |
| GRMZM2G005452 | CC-NBS-LRR | 4:203456994-203463296 | GRMZM2G032751 | CC-NBS-LRR | 10:69613787-69619993 |
| GRMZM2G308064 | CC-NBS-LRR | 4:203637272-203646991 | GRMZM2G060054* | CC-NBS-LRR | 10:83681541-83697115 |
| GRMZM2G006780 | CC-NBS-LRR | 4:203709872-203712302 | GRMZM2G142680 | CC-NBS-LRR | 10:94979016-94982685 |
| GRMZM2G167049* | CC-NBS-LRR | 4:203818715-203824772 | GRMZM2G098677 | CC-NBS-LRR | 10:97329693-97333952 |
| GRMZM2G132403* | TIR-NBS-LRR | 4:218124684-218127963 | GRMZM2G098697* | CC-NBS-LRR | 10:97338667-97343212 |
| GRMZM2G051502 | CC-NBS-LRR | 4:214300902-214309856 | GRMZM2G397785 | CC-NBS-LRR | 10:97377587-97383756 |
| GRMZM2G474092* | CC-NBS-LRR | 4:232498917-232502424 | GRMZM2G397788 | CC-NBS-LRR | 10:97386757-97389185 |
| GRMZM2G454334 | CC-NBS-LRR | 5:31441164-31443177 | GRMZM2G099172* | CC-NBS-LRR | 10:97395092-97396148 |
| GRMZM2G012933* | CC-NBS-LRR | 5:39912904-39914439 | GRMZM2G097813* | CC-NBS-LRR | 10:134993615-134995534 |
| GRMZM2G162098 | CC-NBS-LRR | 5:55459467-55463724 |  |  |  |

*: Newly identified genes in this study.

**Table 2. LRR-RLK encoding genes in maize genome.**

| **Gene** | **Chr. Location** | **Gene** | **Chr. Location** | **Gene** | **Chr. Location** | **Gene** | **Chr. Location** |
| --- | --- | --- | --- | --- | --- | --- | --- |
| GRMZM2G371137 | 1: 5803454-5808250 | GRMZM2G463580 | 3: 16580753-16584193 | GRMZM2G177535 | 5: 51452391-51455712 | GRMZM2G017409 | 7: 163818460-163821972 |
| GRMZM2G009818 | 1: 9800492-9804655 | GRMZM2G022897 | 3: 19770162-19785650 | GRMZM2G177570 | 5: 51462007-51465569 | GRMZM2G009538 | 7: 164988124-164993149 |
| GRMZM2G153393 | 1: 17640006-17655596 | GRMZM2G309897 | 3: 21570405-21573022 | GRMZM2G300133 | 5: 61698661-61702214 | GRMZM2G121573 | 7: 165855165-165860167 |
| GRMZM2G038165 | 1: 26120398-26128604 | GRMZM2G456669 | 3: 88399948-88403516 | GRMZM2G060583 | 5: 64802785-64807893 | GRMZM2G081857 | 7: 167142415-167147144 |
| GRMZM2G043584 | 1: 30258404-30262357 | GRMZM2G391794 | 3: 91614917-91618846 | GRMZM2G168603 | 5: 68680529-68684140 | GRMZM2G080270 | 7: 173321321-173326383 |
| GRMZM5G886952 | 1: 38791249-38799875 | GRMZM2G080503 | 3: 92276172-92279783 | GRMZM5G854880 | 5: 69484758-69487880 | GRMZM2G123314 | 8: 14843475-14846908 |
| GRMZM2G066274 | 1: 46410399-46413363 | GRMZM2G147857 | 3: 93326318-93329971 | GRMZM2G104425 | 5: 81113414-81119465 | GRMZM2G421669 | 8: 14848451-14851761 |
| GRMZM2G023715 | 1: 54273153-54278655 | GRMZM2G409893 | 3: 100262600-100300288 | GRMZM2G060857 | 5: 85586529-85593450 | GRMZM2G463493 | 8: 17060563-17064940 |
| GRMZM2G119850 | 1: 56075120-56079122 | GRMZM2G164502 | 3: 143830339-143833562 | GRMZM2G103070 | 5: 100723578-100737252 | GRMZM2G067675 | 8: 17304016:17309182 |
| GRMZM2G084587 | 1: 76878185-76889886 | GRMZM2G138338 | 3: 147442361-147446195 | GRMZM2G306771 | 5: 123289050-123293128 | GRMZM2G127687 | 8: 26863212-26867537 |
| GRMZM2G002515 | 1: 113534938-113538490 | GRMZM2G447447 | 3: 161622655-161626810 | GRMZM2G146794 | 5: 146875550-146880303 | GRMZM2G070520 | 8: 29734575-29738266 |
| GRMZM2G337766 | 1: 120370797-120374217 | GRMZM2G167253 | 3: 172418169-172421682 | GRMZM2G176206 | 5: 151922862-151926238 | GRMZM2G073884 | 8: 80765150-80768661 |
| GRMZM2G004572 | 1: 141080538-141086039 | GRMZM2G145753 | 3: 184727722-184731545 | GRMZM2G115420 | 5: 176261637-176267226 | GRMZM2G151738 | 8: 89697764-89700024 |
| GRMZM2G429759 | 1: 158703578-158708645 | GRMZM2G465771 | 3: 185367968-185371929 | GRMZM2G026943 | 5: 184479339-184482987 | GRMZM2G080851 | 8: 112421976-112428528 |
| GRMZM2G350793 | 1: 169756325-169759774 | GRMZM2G069201 | 3: 185481180-185489362 | GRMZM2G080537 | 5: 186992292-186995886 | GRMZM2G050548 | 8: 141765271-141771284 |
| GRMZM2G093809 | 1: 180006011-180009342 | GRMZM5G815009 | 3: 187279864-187284817 | GRMZM2G019317 | 5: 204644182-204648555 | GRMZM2G059497 | 8: 149851364-149863364 |
| GRMZM2G461278 | 1: 180366146-180370208 | GRMZM2G177883 | 3: 199347795-199351308 | GRMZM2G463904 | 5: 211800909-211813877 | GRMZM2G360219 | 8: 152127054-152130466 |
| GRMZM2G132763 | 1: 187621125-187624571 | GRMZM2G078926 | 3: 209624891-209634088 | GRMZM2G125081 | 6: 5162305-5165886 | GRMZM2G316907 | 8: 152240805-152244210 |
| GRMZM2G422373 | 1: 202194852-202197410 | GRMZM2G339540 | 3: 224241290-224255475 | GRMZM2G319307 | 6: 6527587-6531419 | GRMZM2G048294 | 8: 154615633-154619705 |
| GRMZM2G428554 | 1: 210641732-210645615 | GRMZM2G439799 | 3: 230338660-230342177 | GRMZM2G700905 | 6: 12617056-12633088 | GRMZM2G059117 | 8: 157002878-157006630 |
| GRMZM2G151955 | 1: 211306417-211312057 | GRMZM2G389299 | 3: 231363799-231371619 | GRMZM2G025105 | 6: 43813330-43814445 | GRMZM2G107484 | 8: 161739951-161757351 |
| GRMZM2G395348 | 1: 215491948-215494092 | GRMZM2G048801 | 4: 4093767-4097080 | GRMZM2G474777 | 6: 88899974-88903683 | AC218972.3_FG004 | 8: 170198252-170201068 |
| GRMZM2G136353 | 1: 220109000-220112377 | GRMZM2G122873 | 4: 5241731-5244116 | GRMZM2G100858 | 6: 91599104-91604620 | AC203173.3_FG002 | 8: 174739279-174742892 |
| GRMZM2G001812 | 1: 233516803-233520836 | GRMZM2G453672 | 4: 5371944-5375255 | GRMZM2G162531 | 6: 97773823-97791863 | GRMZM2G428370 | 9: 12603133-12606224 |
| GRMZM2G001845 | 1: 233584095-233619422 | GRMZM5G867798 | 4: 6103394-6110202 | GRMZM2G171114 | 6: 103870389-103873917 | GRMZM2G127990 | 9: 12623783-12627068 |
| GRMZM2G107872 | 1: 25423809-25428035 | GRMZM2G113373 | 4: 9857198-9864697 | GRMZM2G449817 | 6: 104839663-104842894 | GRMZM2G082855 | 9: 24074354-24081173 |
| GRMZM2G319281 | 1: 258035280-258037705 | GRMZM2G011896 | 4: 10038766-10044977 | GRMZM2G012685 | 6: 107149181-107152477 | GRMZM2G145720 | 9: 35179613-35185362 |
| GRMZM2G137788 | 1: 268802186-268806287 | GRMZM2G068398 | 4: 10223589-10229102 | GRMZM5G809695 | 6: 108369697-108377055 | GRMZM2G046316 | 9: 61459900-61462410 |
| GRMZM2G155312 | 1: 275207652-275217231 | GRMZM2G104384 | 4: 17311139-17323011 | GRMZM2G349665 | 6: 116081434-116084931 | GRMZM2G350918 | 9: 74480747-74484663 |
| AC235547.1_FG005 | 1: 282473696-282477456 | GRMZM2G162829 | 4: 23004196-23007767 | GRMZM2G131609 | 6: 129696721-129700755 | GRMZM2G148702 | 9: 89654357-89658343 |
| GRMZM2G072569 | 1: 282595023-282599365 | GRMZM2G389948 | 4: 23214919-23218326 | GRMZM2G436730 | 6: 130024213-130025681 | GRMZM2G009995 | 9: 89736160-89741950 |
| GRMZM2G021742 | 1: 298511319-298515696 | GRMZM2G122717 | 4: 27843728-27845162 | GRMZM2G149201 | 6: 130782221-130784726 | GRMZM2G141288 | 9: 97192454-97196465 |
| GRMZM2G119759 | 2: 899575-904557 | GRMZM2G039934 | 4: 34834525-34837933 | GRMZM2G121565 | 6: 147401419-147404841 | GRMZM2G071573 | 9: 98256956-98262572 |
| GRMZM2G452142 | 2: 2226789-2230600 | GRMZM2G011806 | 4: 61742558-61747904 | GRMZM2G128315 | 6: 154358844-154362599 | GRMZM2G161664 | 9: 133799373-133804572 |
| GRMZM2G055844 | 2: 5379670-5385668 | GRMZM2G438007 | 4: 65769675-65773352 | AC214817.3_FG004 | 6: 154514150-154517066 | GRMZM2G479243 | 9: 140610164-140617939 |
| GRMZM2G080041 | 2: 9444798-9449014 | GRMZM2G141355 | 4: 66755686-66758822 | GRMZM2G089461 | 6: 154565943-154575228 | GRMZM2G046729 | 9: 146340642-146344213 |
| GRMZM2G337532 | 2: 9927983-9948446 | GRMZM2G150024 | 4: 124660672-124667062 | GRMZM2G174585 | 6: 154839078-154847341 | AC233893.1_FG006 | 9: 146407378-146411588 |
| GRMZM2G114276 | 2: 16005190-16009431 | GRMZM2G071396 | 4: 138248437-138252721 | GRMZM2G132212 | 6: 160430035-160433640 | GRMZM2G045981 | 9: 146683625-146687407 |
| GRMZM2G469313 | 2: 19390329-19392986 | GRMZM2G109830 | 4: 138489006-138492593 | GRMZM2G156905 | 6: 168167529-168170074 | GRMZM2G028643 | 9: 150252956-150255690 |
| GRMZM2G082191 | 2: 31215125-31220469 | GRMZM2G040508 | 4: 138657355-138661148 | GRMZM2G120574 | 7: 1295724-1299401 | GRMZM2G330907 | 9: 150263840-150275563 |
| GRMZM2G021619 | 2: 36708164-36710640 | GRMZM2G702599 | 4: 139022453-139031123 | GRMZM5G852776 | 7: 1988108-1990545 | GRMZM2G009770 | 9: 152025490-152029185 |
| GRMZM2G394321 | 2: 41873087-41875640 | GRMZM2G039665 | 4: 139156285-139159606 | GRMZM2G141517 | 7: 4082077-4086056 | GRMZM2G438840 | 9: 152523091-152526601 |
| GRMZM2G125263 | 2: 72520769-72523282 | GRMZM2G042181 | 4: 139467630-139469392 | GRMZM2G426156 | 7: 4115377-4120421 | GRMZM2G172014 | 9: 153777111-153781389 |
| AC235540.1_FG002 | 2: 121067602-121071772 | GRMZM2G065021 | 4: 149771665-149773464 | GRMZM5G860810 | 7: 8051536-8056829 | GRMZM5G839644 | 10: 6940302-6943942 |
| GRMZM2G073928 | 2: 161907176-161910550 | GRMZM2G150930 | 4: 165794972-165797318 | GRMZM2G432642 | 7: 18436556-18440330 | GRMZM2G002950 | 10: 9785051-9792596 |
| GRMZM2G163724 | 2: 164310419-164313687 | GRMZM2G091632 | 4: 183380469-183386224 | GRMZM2G382273 | 7: 28278050-28283085 | GRMZM5G851140 | 10: 9990889-9995860 |
| GRMZM2G433150 | 2: 173184903-173188005 | GRMZM2G089819 | 4: 183879630-183883191 | GRMZM2G072868 | 7: 50383762-50386524 | GRMZM2G059214 | 10: 11811061-11816472 |
| GRMZM2G002569 | 2: 182995589-182999190 | GRMZM2G039431 | 4: 197994710-197998218 | GRMZM2G092604 | 7: 79119386-79123848 | GRMZM2G002542 | 10: 11907530-11909688 |
| AC233861.1_FG001 | 2: 189738061-189741584 | GRMZM2G100234 | 4: 226544149-226547671 | GRMZM2G110685 | 7: 89116752-89120202 | GRMZM2G472703 | 10: 36254235-36258217 |
| GRMZM2G172429 | 2: 201545881-201550118 | GRMZM2G017022 | 4: 228425476-228427703 | GRMZM2G163860 | 7: 89912626-89957628 | GRMZM2G012176 | 10: 38482153-38487572 |
| GRMZM2G162781 | 2: 213192905-213197565 | GRMZM2G450937 | 4: 237851280-237857810 | GRMZM5G898887 | 7: 95123831-95147199 | GRMZM2G384439 | 10: 71477986-71484046 |
| GRMZM2G169681 | 2: 219686483-219692413 | GRMZM2G451007 | 4: 237861330-237864849 | GRMZM2G468495 | 7: 103304984-103308972 | GRMZM2G015406 | 10: 88410123-88414175 |
| GRMZM2G349875 | 2: 221419815-221427366 | GRMZM2G322348 | 4: 240249146-240253001 | GRMZM2G084248 | 7: 117574984-117578965 | GRMZM2G034155 | 10: 98377679-98381631 |
| GRMZM2G034572 | 2: 237789995-237798040 | GRMZM2G158359 | 5: 10425114-10429156 | GRMZM2G149051 | 7: 130969891-130973647 | GRMZM5G870959 | 10: 121772419-121777860 |
| GRMZM2G012861 | 3: 2763089-2767869 | GRMZM2G178753 | 5: 19545464-19549143 | GRMZM2G313643 | 7: 150075579-150080212 | GRMZM2G016477 | 10: 136139046-136143214 |
| GRMZM2G010693 | 3: 12429859-12434725 | GRMZM2G112309 | 5: 30237719-30241622 | GRMZM2G163138 | 7: 150934423-150944692 | GRMZM2G167280 | 10: 126603529-126608907 |
| GRMZM2G463574 | 3: 16573593-16577181 | GRMZM2G126161 | 5: 42533252-42537728 | GRMZM2G356076 | 7: 163095802-163099649 |  |  |

**Supplementary Material 2.**

**The sequences used for phylogenetic tree building**

>Zea_mays-Cf5-NP_001132758[GRMZM2G107872]

MAAASFARALCLHMLLLFLVVGSARCKTVKRDVKALNEIKSSLGWRVVYSWVGDDPCGHGDLPPWSGVTCSQQGDYRVVTELEVYAVSIVGPFPTAVTNLLDLRRLDLHNNKLTGPIPPQIGRLKHLRILNLRWNKLQDVLPPEIGELKKLTHLYLSFNNFKGEIPVELANLPELRYLYLHENRFTGRIPPELGTLKNLRHLDVGNNHLTGTLRDFIGNGNGFPSLRNLYLNNNELTGVLPDQIANLTNLEILHLSNNKMIGSISPKLVHIPRLIYLYLDNNNFIGRIPEGLYRHPFLKELYIEGNHFRPGTRSKGTHKVLELPDADILV

> Arabidopsis_ thaliana-Cf5-NP_200932

MASRCELLLICVFSLLIAFAHSKTLKRDVKALNEIKASLGWRVVYSWVGDDPCGDGDLPPWSGVTCSTQGDYRVVTELEVYAVSIVGPFPIAVTNLLDLTRLDLHNNKLTGPIPPQIGRLKRLKVLNLRWNKLQDVIPPEIGELKRLTHLYLSFNSFKGEIPKELAALPELRYLYLQENRLIGRIPAELGTLQNLRHLDVGNNHLVGTIRELIRFDGSFPALRNLYLNNNYLSGGIPAQLSNLTNLEIVYLSYNKFIGNIPFAIAHIPKLTYLYLDHNQFTGRIPDAFYKHPFLKEMYIEGNMFKSGVNPIGTHKVLEVSDADFAV

> Oryza__sativa_Japonica_Group-Cf5-NP_001049352

MAAAAAAPRFLPVRPLFLLLLLLVLAGVASGKTVKRDVKALNEIKSSLGWRVVYSWVGDDPCGHGDLPPWSGVTCSQQGDYRVVTELEVYAVSIVGPFPTAVTNLLDLKRLDLHNNKLTGPIPPQIGRLKHLRILNLRWNKLQDVLPPEIGELKKLTHLYLSFNNFKGEIPVELANLPELRYLYLHENRFTGRIPPELGTLKNLRHLDVGNNHLIGTLRDLIGNGNGFPSLRNLYLNNNDLTGVLPDQIANLTNLEILHLSNNRLIGSISPKLVHIPRLTYLYLDNNNFIGRIPEGLYKHPFLKELYIEGNQFRPGSKSKGTHKVLELPDADILV

>Sorghum_bicolor-Cf5-XP_002465654

MAAASFARALSLHVLLLFLLAGSALGKTVKRDVKALNEIKSSLGWRVVYSWVGDDPCGHGDLPPWSGVTCSQQGDYRVVTELEVYAVSIVGPFPTAVTNLLDLRRLDLHNNKLTGPIPPQIGRLKHLRILNLRWNKLQDVLPPEIGELKKLTHLYLSFNNFKGEIPVELANLPELRYLYLHENRFTGRIPPELGTLKNLRHLDVGNNHLTGTLRDFIGNGNGFPSLRNLYLNNNELTGVLPDQIANLTNLEILHLSNNKMIGSISPKLVHIPRLIYLYLDNNNFIGRIPEGLYRHPFLKELYIEGNHFRPGTRSKGTHKVLELPDADILV

> Setaria_italic-Cf5-XP_004985227

MAAARALSLLVVLLLLAGAARGKTVKRDVKALNEIKSSLGWRVVYSWVGDDPCGHGSLPPWSGVTCSQQGDYRVVTELEVYAVSIVGPFPTAVTNLLDLRRLDLHNNKLTGPIPPQIGRLKHLRILNLRWNKLQDVLPPEIGELKKLTHLYLSFNNFKGEIPVELANLPELRYLYLHENRFTGRIPPELGTLKNLRHLDVGNNHLTGTLRDLISNGNGFPSLRNLYLNNNELTGVLPDQIANLTNLEILHLSNNKMIGSISPKLVQIPRLIYLYLDNNNFIGRIPEGLYKHPFLKELYIEGNHFRPGTRSKGTHKVLELPDADILV

>Brachypodium_distachyon-Cf5-XP_003558509

MAAASFARSLPLLFLVLLLAGAARGKTVKRDVKALNEIKSSLGWRVVYSWVGDDPCGHGDLPPWSGVTCSQQGDYRVVTELEVYAVSIVGPFPTAVTNLLDLKKLDLHNNKLTGPIPPQIGRLRHLKILNLRWNKLQDVLPPEIGELKKLTHLYLSFNNFKGEIPVELANLPELRYLYLHQNRFTGRIPPELGTLNHLRHLDVGSNHLIGTLRDVIGIGNGFPSLRNLYVNNNQLIGVLPDQIANLTNLEILHLSNNRLIGSISPRLVHIPRLTYLYLDNNNFIGRIPEGLYKHPFLKELYIEGNQFRPGTRSKGMHKVLELPEADILV

> Zea_ mays - FLS2- XP_008668880[GRMZM2G080041]

MASWKNTCSYSYTPVSNAVAMLALALLVLAAPAASAVPDASASVHLEALLAFKKAVTADPNGTLTSWTVGSGGGGGGGRYPQHCNWTGVACDGAGHVTSIELVDTGLRGTLTPFLGNISTLQLLDLTSNRFGGGIPPQLGRLDGLEGLVLGANNLTGAIPPELGGLGSLQLLDLSNNTLRGGIPRRLCNCSAMAGLSVFNNDLTGAVPDCIGDLTNLNELVLSLNSLDGELPPSFARLTRLETLDLSGNQFSGPIPPGIGNFSRLNIVHMFENRFSGAIPPEIGRCKNLTTLNVYSNRLTGAIPSELGELASLKVLLLYGNALSSEIPRSLGRCASLVSLQLSMNQLTGSIPAELGELRSLRKLMLHANRLTGEVPASLMDLVNLTYLSFSYNSLSGPLPANIGSLQNLQVLVIQNNSLSGPIPASIANCTSLYNASMGFNEFSGPLPAGLGQLQNLHFLSLADNDKLSGDIPEDLFDCSNLRTLTLAGNSFTGSLSPRVGRLSELSLLQLQGNALSGAIPEEMGNLTKLIALQLGGNGFVGRVPKSISNLSSLQKLTLQQNRLDGALPDEIFGLRQLTVLSVASNRFVGPIPDAVSNLRSLSFLDMSNNALNGTVPAAVGSLDHLLTLDLSHNRLAGAIPSALIAKLSALQMYLNLSNNGFTGPIPTEIGALTMVQSIDLSNNRLSGGVPSTLAGCKNLYSLDLSANNLTGALPAGLFPHLDVLTSLNISGNELDGDIPSNIGALKNIQTLDASRNAFTGALPSALANLTSLRSLNLSWNQFEGPVPDSGVFSNLSMSSLQGNAGLCGWKLLAPCRHGGKKGFSRTGLAVLVVLLVLAVLLLLVLVTILFLGYRRYKKKGGSTGANSFAEDFVVPELRKFTCSELDAATSSFDEGNVIGSSNLSTVYKGVLVEPDGKVVAVKRLNLAQFPAKSDKCFLTELATLSRLRHKNLARVVGYACEPGKIKAVVLEFMDNGDLDGAIHGPGRDAQRWTVPERLRACVSVAHGLAYLHTGYDFPIVHCDVKPSNVLLDSDWEARVSDFGTARMLGVHLTDAAAQSATSSAFRGTIGYMAPEFAYMRTVSAKVDVFSFGVLMMELFTKRRPTGMIEEEGVPLTLQQYVDNAISRGLDGVLDVLDPDLKVVTEGDLSTVADVLSLALSCAASDPADRPDMDSVLSALLKMSKVCGRD

> Sorghum_bicolor-FLS2-Sb06g028760

MASCKNTCSCFPLSNVAAVLAIAVLVLAAPAAAAVPDASESVHLEALLAFKEAVTADPNGTLSSWTVGTGNGRGGGGGFPPHCNWTGVACDGAGHVTSIELAETGLRGTLTPFLGNITTLRMLDLTSNRFGGAIPPQLGRLDELKGLGLGDNSFTGAIPPELGELGSLQVLDLSNNTLGGGIPSRLCNCSAMTQFSVFNNDLTGAVPDCIGDLVNLNELILSLNNLDGELPPSFAKLTQLETLDLSSNQLSGPIPSWIGNFSSLNIVHMFENQFSGAIPPELGRCKNLTTLNMYSNRLTGAIPSELGELTNLKVLLLYSNALSSEIPRSLGRCTSLLSLVLSKNQFTGTIPTELGKLRSLRKLMLHANKLTGTVPASLMDLVNLTYLSFSDNSLSGPLPANIGSLQNLQVLNIDTNSLSGPIPASITNCTSLYNASMAFNEFSGPLPAGLGQLQNLNFLSLGDNKLSGDIPEDLFDCSNLRTLDLAWNSFTGSLSPRVGRLSELILLQLQFNALSGEIPEEIGNLTKLITLPLEGNRFAGRVPKSISNMSSLQGLRLQHNSLEGTLPDEIFGLRQLTILSVASNRFVGPIPDAVSNLRSLSFLDMSNNALNGTVPAAVGNLGQLLMLDLSHNRLAGAIPGAVIAKLSTLQMYLNLSNNMFTGPIPAEIGGLAMVQSIDLSNNRLSGGFPATLARCKNLYSLDLSANNLTVALPADLFPQLDVLTSLNISGNELDGDIPSNIGALKNIQTLDASRNAFTGAIPAALANLTSLRSLNLSSNQLEGPVPDSGVFSNLSMSSLQGNAGLCGGKLLAPCHHAGKKGFSRTGLVVLVVLLVLAVLLLLLLVTILFLGYRRYKKKGGSTRATGFSEDFVVPELRKFTYSELEAATGSFDEGNVIGSSNLSTVYKGVLVEPDGKVVAVKRLNLAQFPAKSDKCFLTELATLSRLRHKNLVRVVGYACEPGKIKALVLDFMDNGDLDGEIHGTGRDAQRWTVPERLRACVSVAHGVVYLHTGYDFPVVHCDVKPSNVLLDSDWEARVSDFGTARMLGVHLTDAAAQSATSSAFRGTVGYMAPEFAYMRTVSPKADVFSFGVLMMELFTKRRPTGTIEENGVPLTLQQYVDNAISRGLDGVLDVLDPDMKVVTEGELSTAVDVLSLALSCAAFEPADRPDMDSVLSTLLKMSKVCGGD

> Brachypodium_distachyon-FLS2-BRADI5G21960

MVPPKKTRDASVLPLHVLAFLALAALLGSSPRAAAASASVQLEALLEFKKGVTADPLGALSGWQKKADSRNAIAAAAIVPPPHCNWTGIACNIAGQVTSIQLLESQLEGTLTPFLGNITTLQVLDLTSNAFFGLIPPELGRLQSLEGLILTVNTFTGVIPTSLGALTSLQILDLSNNSLHGTIPSQLCNCSAMWALGLEANNLTGQIPPCIGDLSNLEIFQAYINSLSGELPRSFANLTKLTTLDLSGNQLSGRVPPAIGTFSGLKILQLFENRFSGKIPPELGNCKNLTLLNIYSNRFTGAIPRELGGLTNLKALRVYDNALSSTIPSSLRRCSSLLALGLSMNELTGNIPPELGELRSLQSLTLHENRLTGTVPKSLTRLVNLMRLSFSDNSLSGPLPEAIGSLRNLQVLIIHGNSLSGPIPASIVNCTSLSNASMAFNGFSGSLPAGLGRLQSLVFLSLGDNSLEGTIPEDLFDCVRLRTLNLAENNLTGRLSPRVGKLGGELRLLQLQGNALSGSIPDEIGNLTRLIGLTLGRNKFSGRVPGSISNLSSSLQVLDLLQNRLSGALPEELFELTSLTVLTLASNRFTGPIPNAVSKLRALSLLDLSHNMLNGTVPAGLSGGHEQLLKLDLSHNRLSGAIPGAAMSGATGLQMYLNLSHNAFTGTIPREIGGLAMVQAIDLSNNELSGGVPATLAGCKNLYTLDISSNSLTGELPAGLFPQLDLLTTLNVSGNDFHGEILPGLAGMKHLQTVDVSRNAFEGRVPPGMEKMTSLRELNLSWNRFEGPVPDRGVFADIGMSSLQGNAGLCGWKKLLAPCHAAAGNQRWFSRTGLVTLVVLLVFALLLLVLVVAILVFGHRRYRKKKGIESGGHVSSETAFVVPELRRFTYGELDTATASFAESNVIGSSSLSTVYKGVLVDGKAVAVKRLNLEQFPAMSDKSFLTELATLSRLRHKNLARVVGYAWEREAAGNGNGNRMMKALVLEYMDNGDLDAAIHGGGRGALDAHTAPPRWATVAER

> Triticum_aestivum-FLS2- 6E8764762

MLLKSTRQFIRFDHISTCDSSLKRTKIPREMVARHTWPLPLVLAVLAAAMLAAPPAVADTSAPVHLEALLAFKKGVTADPLGALSDWTIGAGDAARGGVPRHCNWTGVACDGAGRVTSIQLLQTQLQGALTPFLGNISTLQLLDLTENGFTGAIPPQLGRLGELQQLVLPGNGFAGGIPPELGDLGSLQLLDLGNNSLSGGIPSRLCNCSAMWALGLDTNNLTGQIPSCIGDLDQLQIFEAFMNNLDGELPPSFAKLTQMKSLDLSANKLSGSIPPEIGNFSHLWILQLLENRFSGAIPPELGRCKNLTMLSIYSNRFTGAIPRELGELVNLEHLHLYDNALSSTIPSSLGRCTSLVALGLSMNQLTGSIPPELGELRSLQTLTLHANRLTGTVPTSLTNLVNLTYLSLNQNSLSGRLPENIGSLRNLQKLVIHNNSLSGPIPASIANCTLLSNASMSNNEFTGHLPAGLGRLKDLAFLSVGINSLTGGIPEDLFDCGSLRTLDLAWNNFTGALNRRVGQLSELRRLHLQWNALSGTIPEEIGNLTNLIDLKLGWNRFAGRVPASISNISSSLQVLDLSHNRLNGVLPDELFELRQLTILNLASNRFAGPIPAAVSKLQSLSLFDLSKNRLNGTFPAGLGGHEQLLTLDLSHNRLSGAIPGAAIAAMSTVQMYLNLSNNAFTGPIPREVGGLTMAQAIDLSNNQLSGGIPATLAGCKNLYSLDLSANNLVGTLPAGLFPQLDLLTSLNVSHNDLDGEINPDMAALKHIQKLDLSSNAFGGTIPPALANLTSLRELNLSSNHLEGPVPDTGVFRNLSVSSLQGNAGLCGWKLLAPCHAAAAGKPRFSRTKLVVLVVLLVLALLLLFSLVTILIVGCRRYKKKMVKSDGSSHLSETFVVPELRRFTYGELEAATGSFDQGNVIGSSSLSTVYKGVLVEPDGKAVAVKRLNLEQFPAMSDKSFLTELVTLSRLRHKNLARVVGYAWEAFKMKALVLEYMDNGDLDGAIHGPDAPRWTVAERLRVCVSVAHGLVYLHSGYGFPIVHCDVKPSNVLLDADWEARVSDFGTARMLGVHLTEAAAPDSATSSAFRGTVGYMAPELAYMRGASPKADVFSFGVMVMELFTKRRPTGNIEEDGVPMTLQQRVGNALSSGLEGVAGVLDPGMKVATEIDLSTAADALRLASSCTEFEPADRPDMNGVLSALLKMSRACGAE

>Triticum_urartu-FLS2-EMS63184

MWALGVDINNLTGQIPSCIGDLDKLQIFEAFMNNLDGELPPSFAKLTQMKSLDLSANKLSGSIPQEIGNFSHLWILQMWENRFSGPIPSELGRFGANSLTGGIPEDLFDCGSLRTLDLAWNNFTGGLNRRVGQLGELRRLHLQWNALSGTIPEEIGNLTNLIDLKLGGNRFAGRIPASISNMSSSLQVLDLSHNRLHGALPAELFELRQPTILDLGSNRCAGAIPAEVSNLRSLSFLDLSKNRLNGTFPAGLGGHEQLLTLDLSHNRLSGAIPGAAVAAMSTVQMYLNLSNNAFTGPIPREVGGLTMVQAIDLSNNQLSGGIPATLAGCKNLYSLDLSANNLVGTLPAGLFPQLDLLTTLNVSHNDLDGEINPDMAALKHIQTLDLSSNAFAGTIPPALANLTSLRELNLSSNHLEGPVPDTGVFRNLSVSSLQGNPGLCGWNLLAPCHAAGAGKPRFSRTGLVVLVVLLVLALLLLFSLVTILVVCCRRYKKKRVKSDGSSHLSETFVVPELRRFTYGELESATGSFDQGNVIGSSSLSTVYKGVLVEPDGKAVAVKRLNLEQFPAMSDKSFLTELATLSRLRHKNLARVVGYAWEAFKMKALVLEYMDNGDLDGAIHGPDAPRWTVAERLRVCVSVAHGLVYLHSGYGFPLVHCDLAYMRGASPKADVFSFGVLVMELFTKRRPTGNIEEDGVPMTLQQLVGNALSRGLEGVAGVLDSGMKVATEIELSTAADALRLASSCAEFEPADRPDMNGVLSALLKMSRACGGD

> Oryza_sativa_Indica_Group-FLS2-CAH68341

MSQHYTKTICIAVVLVAVLFSLSSSAAAGSGAAVSVQLEALLEFKNGVADDPLGVLAGWRVGKSGDGAVRGGALPRHCNWTGVACDGAGQVTSIQLPESKLRGALSPFLGNISTLQVIDLTSNAFAGGIPPQLGRLGELEQLVVSSNYFAGGIPSSLCNCSAMWALALNVNNLTGAIPSCIGDLSNLEIFEAYLNNLDGELPPSMAKLKGIMVVDLSCNQLSGSIPPEIGDLSNLQILQLYENRFSGHIPRELGRCKNLTLLNIFSNGFTGEIPGELGELTNLEVMRLYKNALTSEIPRSLRRCVSLLNLDLSMNQLAGPIPPELGELPSLQRLSLHANRLAGTVPASLTNLVNLTILELSENHLSGPLPASIGSLRNLRRLIVQNNSLSGQIPASISNCTQLANASMSFNLFSGPLPAGLGRLQSLMFLSLGQNSLAGDIPDDLFDCGQLQKLDLSENSFTGGLSRRVGQLGNLTVLQLQGNALSGEIPEEIGNLTKLISLKLGRNRFAGHVPASISNMSSLQLLDLGHNRLDGMFPAEVFELRQLTILGAGSNRFAGPIPDAVANLRSLSFLDLSSNMLNGTVPAALGRLDQLLTLDLSHNRLAGAIPGAVIASMSNVQMYLNLSNNAFTGAIPAEIGGLVMVQTIDLSNNQLSGGVPATLAGCKNLYSLDLSGNSLTGELPANLFPQLDLLTTLNISGNDLDGEIPADIAALKHIQTLDVSRNAFAGAIPPALANLTALRSLNLSSNTFEGPVPDGGVFGNLTMSSLQGNAGLCGGKLLVPCHGHAAGNKRVFSRTGLVILVVLIALSTLLLLMVATILLIGYRRYRRKRRAAGIAGDSSEAAVVVPELRRFSYGQLAAATNSFDQGNVIGSSNLSTVYKGVLAGDADGGMVVAVKRLNLEQFPSKSDKCFLTELATLSRLRHKNLARVVGYAWEAGKIKALVLDYMVNGDLDGAIHGGAAAPPTAPSRWTVRERLRVCVSVAHGLVYLHSGYDFPVVHCDVKPSNVLLDGDWEARVSDFGTARMLGVHLPAAADAAAQSTATSSAFRGTVGYMAPEFAYMRTVSTKVDVFSFGVLAMELFTGRRPTGTIEEDGVPLTLQQLVDNAVSRGLDGVHAVLDPRMKVATEADLSTAADVLAVALSCAAFEPADRPDMGAVLSSLLKMSKLVGED

> Oryza_sativa_Japonica_Group-FSL2-CAE02151

MSQHYTKTICIAVVLVAVLFSLSSAAAAGSGAAVSVQLEALLEFKNGVADDPLGVLAGWRVGKSGDGAVRGGALPRHCNWTGVACDGAGQVTSIQLPESKLRGALSPFLGNISTLQVIDLTSNAFAGGIPPQLGRLGELEQLVVSSNYFAGGIPSSLCNCSAMWALALNVNNLTGAIPSCIGDLSNLEIFEAYLNNLDGELPPSMAKLKGIMVVDLSCNQLSGSIPPEIGDLSNLQILQLYENRFSGHIPRELGRCKNLTLLNIFSNGFTGEIPGELGELTNLEVMRLYKNALTSEIPRSLRRCVSLLNLDLSMNQLAGPIPPELGELPSLQRLSLHANRLAGTVPASLTNLVNLTILELSENHLSGPLPASIGSLRNLRRLIVQNNSLSGQIPASISNCTQLANASMSFNLFSGPLPAGLGRLQSLMFLSLGQNSLAGDIPDDLFDCGQLQKLDLSENSFTGGLSRLVGQLGNLTVLQLQGNALSGEIPEEIGNMTKLISLKLGRNRFAGHVPASISNMSSLQLLDLGHNRLDGVFPAEVFELRQLTILGAGSNRFAGPIPDAVANLRSLSFLDLSSNMLNGTVPAALGRLDQLLTLDLSHNRLAGAIPGAVIASMSNVQMYLNLSNNAFTGAIPAEIGGLVMVQTIDLSNNQLSGGVPATLAGCKNLYSLDLSGNSLTGELPANLFPQLDLLTTLNISGNDLDGEIPADIAALKHIQTLDVSRNAFAGAIPPALANLTALRSLNLSSNTFEGPVPDGGVFRNLTMSSLQGNAGLCGGKLLAPCHGHAAGKKRVFSRTGLVILVVLIALSTLLLLMVATILLVSYRRYRRKRRAADIAGDSPEAAVVVPELRRFSYGQLAAATNSFDQGNVIGSSNLSTVYKGVLAGDADGGMVVAVKRLNLEQFPSKSDKCFLTELATLSRLRHKNLARVVGYAWEAGKIKALVLDYMVNGDLDGAIHGGAAAPPPAPSRWTVRERLRVCVSVAHGLVYLHSGYDFPVVHCDVKPSNVLLDGDWEARVSDFGTARMLGVHLPAAANAAAQSTATSSAFRGTVGYMAPEFAYMRTVSTKVDVFSFGVLAMELFTGRRPTGTIEEDGVPLTLQQLVDNAVSRGLDGVHAVLDPRMKVATEADLSTAADVLAVALSCAAFEPADRPDMGAVLSSLLKMSKLVGED

>Nicotiana_sylvestris-FLS2-XP_009801818

MSKTVLYALAIFSFTFFIPLSYGQTPSLEIEVAALKAFKNSISDDPFGALVDWTNANHHCNWSGITCDPSSNHVINITLFETQLKGEISPFLGNLSKLQVLDLTLNSFTGNIPPQLGHCTELVELILYENLLSGEIPAELGNLRNLQLIDFGNNFLNGSIPDSICDCTELLLVSLINNSFTGKLPSDIGNLANLQLFVAYENNFIGSIPTSIRKLTALQTLDLSENRLSGPIPPEIGNLSSLETLQLHLNFLSGKIPSELGLCTNLVTLNMYTNQFTGSIPPELGNLENLQTLRLYNNQLNSSIPASLFHLKSLTHLGLSQNELTGQIPPELGSSMSLQVLTLHSNRLSGEIPSTITKLTNLTYLSLSFNLLTGSLPLEFGLLYNLKNLTASNNLLEGSIPSSITNCSHLLVLTLTYNRITGEIPIGLGQLSNLTFLSLGSNKMVGEIPDDFFNCSMLEVLDLSDNNFSGKLKPMIGRLSKLRVLRARTNSFLGPIPPEIGKLSQLMDLVLDENSFSGVIPPDISMLSDLQGLSLYDNKLEGKLPVQLFELKQLNELRLQNNNFLGPIPHQISKMELLSLLDLSGNKLNGTLPESMASLRRLMTIDLSHNLLTGSLPRAVLASMRSMQFYLNVSSNFLNGTIPDEIGVLEMVQEIDMSNNNLSGSIPRSFGRCKNLFSLDLSGNMLSGPAPGAILTKLSELVFLNLSKNRLQSELPEMAGLPHLRSVDLSHNKFKGIIPERFASMPALKYLNLSFNQLEGHIPKGGVFNNIKLEDLLGNPSLCGTKFLRPCSTKSIRTGTHGFSKKTLIILAALGSVFGLILLVLGVFFLNQYMKKQKVKDTEDMIPKYASALSLKRFYQKDLELATDNFSPENIIGASSLSTVYKGRLEDGKIVAVKKLNHRFAAEADKCFDREVRTLSQLRHRNLVKVLGYAWESKKLKALVLEYMEKGNLDNIIYDQMVDDWTLSNRIDILVSVASGLSYLHSGYDFPIVHCDLKPSNILLDENMEAHVSDFGTARMLGIHLQDGSSISSASVFEGTIGYLAPEFAYMRKVTTKVDVFSFGVIVMEIITKRRPTGLTGADELPMTLHQIVRNAAANGINELIQIVDPNLASYVSKKQDVVEGLLKLALSCTSPDPEDRPDMEQVLSSLSKLRKMECMNSHACLVKDAI

>Arabidopsis_thaliana-FLS2-NP_199445

MKLLSKTFLILTLTFFFFGIALAKQSFEPEIEALKSFKNGISNDPLGVLSDWTIIGSLRHCNWTGITCDSTGHVVSVSLLEKQLEGVLSPAIANLTYLQVLDLTSNSFTGKIPAEIGKLTELNQLILYLNYFSGSIPSGIWELKNIFYLDLRNNLLSGDVPEEICKTSSLVLIGFDYNNLTGKIPECLGDLVHLQMFVAAGNHLTGSIPVSIGTLANLTDLDLSGNQLTGKIPRDFGNLLNLQSLVLTENLLEGDIPAEIGNCSSLVQLELYDNQLTGKIPAELGNLVQLQALRIYKNKLTSSIPSSLFRLTQLTHLGLSENHLVGPISEEIGFLESLEVLTLHSNNFTGEFPQSITNLRNLTVLTVGFNNISGELPADLGLLTNLRNLSAHDNLLTGPIPSSISNCTGLKLLDLSHNQMTGEIPRGFGRMNLTFISIGRNHFTGEIPDDIFNCSNLETLSVADNNLTGTLKPLIGKLQKLRILQVSYNSLTGPIPREIGNLKDLNILYLHSNGFTGRIPREMSNLTLLQGLRMYSNDLEGPIPEEMFDMKLLSVLDLSNNKFSGQIPALFSKLESLTYLSLQGNKFNGSIPASLKSLSLLNTFDISDNLLTGTIPGELLASLKNMQLYLNFSNNLLTGTIPKELGKLEMVQEIDLSNNLFSGSIPRSLQACKNVFTLDFSQNNLSGHIPDEVFQGMDMIISLNLSRNSFSGEIPQSFGNMTHLVSLDLSSNNLTGEIPESLANLSTLKHLKLASNNLKGHVPESGVFKNINASDLMGNTDLCGSKKPLKPCTIKQKSSHFSKRTRVILIILGSAAALLLVLLLVLILTCCKKKEKKIENSSESSLPDLDSALKLKRFEPKELEQATDSFNSANIIGSSSLSTVYKGQLEDGTVIAVKVLNLKEFSAESDKWFYTEAKTLSQLKHRNLVKILGFAWESGKTKALVLPFMENGNLEDTIHGSAAPIGSLLEKIDLCVHIASGIDYLHSGYGFPIVHCDLKPANILLDSDRVAHVSDFGTARILGFREDGSTTASTSAFEGTIGYLAPEFAYMRKVTTKADVFSFGIIMMELMTKQRPTSLNDEDSQDMTLRQLVEKSIGNGRKGMVRVLDMELGDSIVSLKQEEAIEDFLKLCLFCTSSRPEDRPDMNEILTHLMKLRGKANSFREDRNEDREV

> Zea_mays--PSKR1-XP_008645956

MEHFLMRGTATWPRRSFLCLSAVLVLLLLSPVDSLKKTTISCDPGDLKALEGFSEALDGGSVAGWEHPNATSCCAWPGVRCDGSGRVVRLDLHGRRLRGELPLSLAQLDQLQWLNLSDNNFHGAVPAPVLQLQRLQRLDLSDNELAGTLLDNMSLPLIELFNISYNNFSGSHPTFRGSERLTAFDAGYNSFSGQINTSICGSSGEISVLRFTSNLFTGDFPAGFGNCTKLEELHVELNSISGRLPDDLFRLPSLKVLSLQENQLTWGMSPRFSNLSSLERLDISFNSFFGHLPNVFGSLRKLEFFSAQSNLFGGPLPPSLCRSPSLKMLYLRNNSLNGEVNLNCSAMTQLSSLDLGTNKFIGTIDSLSDCRNLRSLNLATNNLSGDIPDGFRKLQSLTYLSLSNNSFTDVPSALSVLQNCSSLTSLVLTKNFRDEKALPMTGIHGFHNIQVFVIANSHLSGSVPPWLANFTQLKVLDLSWNQLVGNIPPWIGDLEFLFYLDLSNNSLSGGIPESLSSMKALVTRKVSQESTETDYFPFFIKRNKTGKGLQYNQVSSFPPSLVLSHNRLTGPILSGFGILKNLHVLDLSNNNISGIIPDDLSEMSSLESLDLSHNNLTGGIPSSLTKLNFLSSFSVAYNNLNGTIPSAGQFLTFSSSAYEGNPKLCGIRLGLPRCHPTPAPAIAATNKRKNKGIIFGIAMGVAVGAAFVLSIAAVFVLKSNFRRQDHTVKAVADTDRALELAPASLVLLFQNKADKALTIADILKSTNNFDQANIIGCGGFGIVYKATLQDGAAIAIKRLSGDFGQMEREFKAEVETLSKAQHPNLVLLQGYCRIGSDRLLIYSFMENGSLDHWLHESPDGPSRLIWPRRLQIAKGAARGLAYLHLSCQPHILHRDIKSSNILLDENFEAHLADFGLARLICPYATHVTTDLVGTLGYIPPEYGQSSVATFKGDVYSFGIVLLELLTGKRPIDMCKPKGARELVSWVTLMKKENREADVLDRAMYDKKFETQMRQVIDIACLCVSDSPKLRPLTHQLVMWLDNIGVTSDEPK

> Arabidopsis_ thaliana-PSKR1-NP_178330

MRVHRFCVIVIFLTELLCFFYSSESQTTSRCHPHDLEALRDFIAHLEPKPDGWINSSSSTDCCNWTGITCNSNNTGRVIRLELGNKKLSGKLSESLGKLDEIRVLNLSRNFIKDSIPLSIFNLKNLQTLDLSSNDLSGGIPTSINLPALQSFDLSSNKFNGSLPSHICHNSTQIRVVKLAVNYFAGNFTSGFGKCVLLEHLCLGMNDLTGNIPEDLFHLKRLNLLGIQENRLSGSLSREIRNLSSLVRLDVSWNLFSGEIPDVFDELPQLKFFLGQTNGFIGGIPKSLANSPSLNLLNLRNNSLSGRLMLNCTAMIALNSLDLGTNRFNGRLPENLPDCKRLKNVNLARNTFHGQVPESFKNFESLSYFSLSNSSLANISSALGILQHCKNLTTLVLTLNFHGEALPDDSSLHFEKLKVLVVANCRLTGSMPRWLSSSNELQLLDLSWNRLTGAIPSWIGDFKALFYLDLSNNSFTGEIPKSLTKLESLTSRNISVNEPSPDFPFFMKRNESARALQYNQIFGFPPTIELGHNNLSGPIWEEFGNLKKLHVFDLKWNALSGSIPSSLSGMTSLEALDLSNNRLSGSIPVSLQQLSFLSKFSVAYNNLSGVIPSGGQFQTFPNSSFESNHLCGEHRFPCSEGTESALIKRSRRSRGGDIGMAIGIAFGSVFLLTLLSLIVLRARRRSGEVDPEIEESESMNRKELGEIGSKLVVLFQSNDKELSYDDLLDSTNSFDQANIIGCGGFGMVYKATLPDGKKVAIKKLSGDCGQIEREFEAEVETLSRAQHPNLVLLRGFCFYKNDRLLIYSYMENGSLDYWLHERNDGPALLKWKTRLRIAQGAAKGLLYLHEGCDPHILHRDIKSSNILLDENFNSHLADFGLARLMSPYETHVSTDLVGTLGYIPPEYGQASVATYKGDVYSFGVVLLELLTDKRPVDMCKPKGCRDLISWVVKMKHESRASEVFDPLIYSKENDKEMFRVLEIACLCLSENPKQRPTTQQLVSWLDDV

> Morus_notabilis-PSKR1-EXC37937

MGIQDFWVFLIVLGFCLRIQCQNLTCDQNDLKALRDFMAGLQTSIDGWEEKNPSSNCCKWPGITCENETGKVSVLDLGSKKLTGKLSESLGSLNQLRTLNLSHNYLKSSIPISLFSMSNLQVLDLSFNDFYGAVPDTVNLSSIQYLDMSQNYLNGSLPNHICNGGRSELKILKLAANFFSGDLPSGFGNCTFLNHLCVGMNNLTRISEGVFRLRNISELIIPDNKLSGQLSDGIGNLTNLVRLDISTNEFSGAIPNVFHKLGKLHSFVAHSNKFTGGIPESLTNSPSISLLNVRNNSLVGPININCAAMVNLTSLDLGSNKFNGSISYKLPSCRHLNNINIARNKLVGEIPESYKDFHSLSYFSLSNSSNTNLSSALRILQQCENLTTLVLSLNFHDEELPSDPSFHFEKLRILVIANCRLKGSLPQWLSKSKRLQLLDLSWNNLVGKVPPWLGDFDSLFYLDISNNSFTGEIPENITRLRSLIDREISLEEPSPDFPFFMKRNVSSRGLQYNQVQSFPPTLDLSSNNLSGPIWPEFGNLKKLHVLDLKLNNLSGSIPSNLSGMSSLETLDLSHNMLSGTIPSSLVKLNFLSKFNVAYNKLHGEIPSGGQFATFPNSSFEGNNLCGDHAVPCASNQSLPSHPSSHSTKKRGVVVGLTIGIVFGAALFLSLLFVFVLRKHRPREIDPEREDGYTNDKDLEQLGSRLVVLFQNKENTKELCVDDLLKSTNNFDQANIIGCGGFGLVYRATLPDGKKVAIKRLSGDCGQMEREFRAEVETLSRAQHPNLVLLQGYCIYKNDRLLIYSYMENSSLDYWLHERVDGPAFLKWETRLQIARGAARGLAYLHQSCEPHILHRDIKSSNILLDENFEAHLADFGLARLILPYDTHVTTDLVGTLGYIPPEYGQASVATYKGDVYSFGVVLLELLTGKRPMDMCKPKGCRDLISWVFQMKKEKKESEVFDPFIYNKHNDKELLQILEIACLCLSEFPKLRPTTQQLVSWLDGIDIDI

> Brassica_rapa -PSKR1- XP_009129163

MRVHRLWVIVTFLTELLLCFRPSDSQTPLTCHPRDLNALRDFITNIEPKPQDWLLVNDNCCNWTGITCDNDNNDNTIRVTKLELGNKKLSGTLSESLGNLDQIKVLNLSRNFIKDSIPLSILTSPTLQTLDLSFNDLSGEIPQTLNLPSLQSLDLSSNNFNGSLSRSIGNLSSLARLDVSWNSFSGEIPDVFHELTRLKYLSAQTNRLTGGIPRSLASSRTLNLLNLRNNSLTGPLLLNCTAMVELNSLDLGTNRFNGRLPENLPVCKRLKNVNLARNGFRGEVPESFKNFQSLSYFSLSNSSLVNITSALRILQNCKNLTALVLTLNFNGEALPADDASIVGFEKLKVLVVANCRLTGSVPRWLSSSRDLQLLDLSWNRLTGAIPSWIGDFNSLFYLDLSNNSFTGEIPKSLTQLQSLTSGSISLDEPSPDFPFFMKRNESARGLQYNQIVGFPPTIELGHNNLSGPIWEEFGDLKKLHVFDLKWNELSGPIPSSLSDMTSLELLDLSNNRLSGSIPGSLQNLTFLSKFSVASNNLTGRIPSGGQFQTFPNSSFEFNNLCGEHRFPCSGDASSAGTTTYIKHSRRRSRGGEIGMAVGIAFGSVFLLTLLALIVLRARRRSGEVDPEIEEESMNRKEIEETGSKIVVLFQNNNDNALSYDDLLDSTDNFDQSNIIGCGGFGMVYKAMLPDGRKVAIKRLSGDCGQIEREFKAEVETLSRAQHPNLVLLQGFCFYKTDRLLIYSYMENGSLDYWLHERNDGPALLGWRTRVRIAQGAAKGLYYLHQACEPHILHRDIKSSNILLDENFDSHLADFGLARLMNPYETHVSTDLVGTLGYIPPEYGQASVATYKGDVYSFGVVLLELLTDRRPVDMCKPKGGRDLISWVVRMKSEGRASEVFDPFIHGKENEKEMVRVLEIACLCLSGNPKQRPTTEQLVSWLDHV

> Vitis_vinifera -PSKR1-XP_002273186

MGDSVFWVLTVLIVLQVQVVCSQNQTCSSNDLAVLLEFLKGLESGIEGWSENSSSACCGWTGVSCNSSAFLGLSDEENSNRVVGLELGGMRLSGKVPESLGKLDQLRTLNLSSNFFKGSIPASLFHFPKLESLLLKANYFTGSIAVSINLPSIKSLDISQNSLSGSLPGGICQNSTRIQEINFGLNHFSGSIPVGFGNCSWLEHLCLASNLLTGALPEDLFELRRLGRLDLEDNSLSGVLDSRIGNLSSLVDFDISLNGLGGVVPDVFHSFENLQSFSAHSNNFTGQIPYSLANSPTISLLNLRNNSLSGSININCSVMGNLSSLSLASNQFTGSIPNNLPSCRRLKTVNLARNNFSGQIPETFKNFHSLSYLSLSNSSLYNLSSALGILQQCRNLSTLVLTLNFHGEELPGDSSLQFEMLKVLVIANCHLSGSIPHWLRNSTGLQLLDLSWNHLNGTIPEWFGDFVFLFYLDLSNNSFTGEIPKNITGLQGLISREISMEEPSSDFPLFIKRNVSGRGLQYNQVGSLPPTLDLSNNHLTGTIWPEFGNLKKLNVFELKCNNFSGTIPSSLSGMTSVETMDLSHNNLSGTIPDSLVELSFLSKFSVAYNQLTGKIPSGGQFQTFSNSSFEGNAGLCGDHASPCPSDDADDQVPLGSPHGSKRSKGVIIGMSVGIGFGTTFLLALMCLIVLRTTRRGEVDPEKEEADANDKELEQLGSRLVVLFQNKENNKELCIDDLLKSTNNFDQANIIGCGGFGLVYRATLPDGRKVAIKRLSGDCGQMEREFQAEVEALSRAQHPNLVLLQGYCKYKNDRLLIYSYMENSSLDYWLHEKLDGPSSLDWDTRLQIAQGAAMGLAYLHQSCEPHILHRDIKSSNILLDEKFEAHLADFGLARLILPYDTHVTTDLVGTLGYIPPEYGQASVATYKGDVYSFGVVLLELLTGKRPMDMCKPRGCRDLISWVIQMKKEKRESEVFDPFIYDKQHDKELLRVLDIACLCLSECPKIRPSTEQLVSWLNNILM

>Sorghum_bicolor-PSKR1-XP_002454207

MEHFLMQRTTTWPCRFSLCLSVLVLLLFLSPVDSLNQSSSSCDPGDLKALEGFYKGLDRGIAGWTFPNGTSDAASCCAWLGVTCDGSGKVIGLDLHGRRLRGQLPLSLTQLDQLQWLNLSDNNFGGAVPAPLFQLQRLQQLDLSYNELAGILPDNMSLPLVELFNISYNNFSGSHPTLRGSERLIVFDAGYNSFAGQIDTSICESSGEISVLRFSSNLFTGDFPAGFGNCTKLEELYVELNIISRRLPEDLFRLPSLKILSLQENQLSGGMSPRFGNLSNLDRLDISFNSFSGHIPNVFGSLRKLEFFSAQSNLFRGPLPPSLCHSPSLKMLYLRNNSLNGEINLNCSAMTQLSSLDLGTNKFIGTIYSLSDCRNLKSLNLATNNLSGEIPAGFRKLQSLTYLSLSNNSFTDMPSALSVLQDCPSLTSLVLTKNFHDQKALPMTGIQGFHSIQVFVIANSHLSGPVPPWLANFTQLKVLDLSWNQLTGNIPACIGDLEFLFYLDLSNNSLSGEIPENLSNMKALVTRKISQESTETDYFPFFIKRNKTGKGLQYNQVSSFPPSLVLSHNKLTGPILSGFGILKHLHVLDLSNNNISGTIPDDLSGMSSLESLDLSHNNLTGGIPYSLTKLNFLSSFSVAYNNLNGTIPSGGQFSTFSSSAYEGNPKLCGIRLGLPRCHSTPAPTIAATNKRKNKGIIFGIAMGIAVGAAFILSIAVIFVLKSSFNKQDHTVKAVKDTNQALELAPASLVLLFQDKADKALTIADILKSTNNFDQANIIGCGGFGLVYKATLQDGAAIAIKRLSGDFGQMEREFKAEVETLSKAQHPNLVLLQGYCRIGSDRLLIYSFMENGSLDHWLHEKPDGPSRLIWPRRLQIAKGAARGLAYLHLSCQPHILHRDVKSSNILLDENFEAHLADFGLARLICPYATHVTTDLVGTLGYIPPEYGQSSVATFKGDVYSFGIVLLELLTGKRPVDMCKPKGARELVSWVTHMKKENREADVLDRAMYDKKFETQMIQMIDVACLCISDSPKLRPLTHQLVLWLDNIGVTSDAPK

> Oryza_sativa_Japonica Group-PSKR1-BAD23737

MMQLTTTWPWRFFFCLFFHLLFLFPTNSLNQSYCDPGDASALLGFMQGLSGSGSGWTVPNATSETANCCAWLGVKCNDGGRVIGLDLQGMKLRGELAVSLGQLDQLQWLNLSSNNLHGAVPATLVQLQRLQRLDLSDNEFSGEFPTNVSLPVIEVFNISLNSFKEQHPTLHGSTLLAMFDAGYNMFTGHIDTSICDPNGVIRVLRFTSNLLSGEFPAGFGNCTKLEELYVDLNSITGSLPDDLFRLSSLRDLSLQENQLSGRMTPRFGNMSSLSKLDISFNSFSGYLPNVFGSLGKLEYFSAQSNLFRGPLPSSLSHSPSLKMLYLRNNSFHGQIDLNCSAMSQLSSLDLGTNKFIGTIDALSDCHHLRSLNLATNNLTGEIPNGFRNLQFLTYISLSNNSFTNVSSALSVLQGCPSLTSLVLTKNFNDGKALPMTGIDGFHNIQVFVIANSHLSGSVPSWVANFAQLKVLDLSWNKLSGNIPAWIGNLEHLFYLDLSNNTLSGGIPNSLTSMKGLLTCNSSQQSTETDYFPFFIKKNRTGKGLRYNQVSSFPPSLILSHNMLIGPILPGFGNLKNLHVLDLSNNHISGMIPDELSGMSSLESLDLSHNNLTGSIPSSLTKLNFLSSFSVAFNNLTGAIPLGGQFSTFTGSAYEGNPKLCGIRSGLALCQSSHAPTMSVKKNGKNKGVILGIAIGIALGAAFVLSVAVVLVLKSSFRRQDYIVKAVADTTEALELAPASLVLLFQNKDDGKAMTIGDILKSTNNFDQANIIGCGGFGLVYKATLPDGATIAIKRLSGDFGQMEREFKAEVETLSKAQHPNLVLLQGYCRIGNDRLLIYSYMENGSLDHWLHEKPDGPSRLSWQTRLQIAKGAARGLAYLHLSCQPHILHRDIKSSNILLDEDFEAHLADFGLARLICPYDTHVTTDLVGTLGYIPPEYGQSSVANFKGDVYSFGIVLLELLTGKRPVDMCKPKGARELVSWVLHMKEKNCEAEVLDRAMYDKKFEMQMVQMIDIACLCISESPKLRPLTHELVLWLDNIGGSTEATK

>Zea_ mays-PSY1R-XP_008644981

MQSLNWLCSSSSTTRLHVPSFGIALVVLLSCVSVASSCTDQERSSLIDFRDGLSPDGNGGLHMLWANSTDCCQWEGITCSNDGAVTEVLLPSRGLEGRIPPSLGNLTGLQRLNLSCNSLYGNLPPELVFSSSSSILDVSFNHLSGPLQERQSPISGLPLKVLNISSNFFTGQLSSTALQVMNNLVALNASNNSFAGPLPSSICIHAPSLVTLDLCLNDFSGTISPEFGNCSKLTVLKAGHNNLTGGLPHELFNATSLEHLSFPNNNLQGALDGSSLVKLRNLIFLDLGSNGLEGNMPDSIGQLGRLEELHLDNNLIVGELPSALSNCRSLKYITLRNNSFMGDLSRINFTQMDLTTADFSLNKFNGTIPENIYACSNLIALRLAYNNFHGQFSPRIANLRSLSFLSVTNNSFTNITGALQNLNRCKNLTSLLIGTNFKGETIPQYAAIDGFENLRVLTIDACPLVGEIPIWLSKLTRLEILDLSYNHLTGTIPSWINRLELLFFLDISSNRLTGDIPPELMEMPMLQSEKNSAKLDPKFLELPVFWTQSRQYRLLNAFPNVLNLCNNSLTGIIPQGIGQLKVLNVLNFSTNSLSGEIPQQICNLTNLQTLDVSNNQLTGELPSALSNLHFLSWFNVSNNDLEGPVPSGGQFNTFTNSSYIGNPKLCGPMLSVHCGSVEEPRASMKMRHKKTILALALSVFFGGLAILFLLGRLILSIRSTESADRNKSSNNRDIEATSFNSASEHVRDMIKGSTLVMVPRGKGESNNLTFNDILKATNNFDQQNIIGCGGNGLVYKAELPCGSKLAIKKLNGEMCLMEREFTAEVEALSMAQHENLVPLWGYCIQGNSRLLIYSFMENGSLDDWLHNTDNANSFLDWPTRLKIAQGAGRGLSYIHNTCNPNIVHRDVKSSNILLDREFNAYVADFGLARLILPYNTHVTTELVGTLGYIPPEYGQAWVATLRGDIYSFGVVLLELLTGKRPVQVLTKSKELVQWVREMRSQGKDIEVLDPALRGRGHDEQMLNVLEVAYKCINHNPGLRPTIQEVVYCLETIVEPQQVQVQL

> Arabidopsis_thaliana-PSY1R-NP_177374

MIDEKMRSKSIGPFVRQVKPLSPHMVLFVLLYVLSISVFFLTVSEAVCNLQDRDSLLWFSGNVSSPVSPLHWNSSIDCCSWEGISCDKSPENRVTSIILSSRGLSGNLPSSVLDLQRLSRLDLSHNRLSGPLPPGFLSALDQLLVLDLSYNSFKGELPLQQSFGNGSNGIFPIQTVDLSSNLLEGEILSSSVFLQGAFNLTSFNVSNNSFTGSIPSFMCTASPQLTKLDFSYNDFSGDLSQELSRCSRLSVLRAGFNNLSGEIPKEIYNLPELEQLFLPVNRLSGKIDNGITRLTKLTLLELYSNHIEGEIPKDIGKLSKLSSLQLHVNNLMGSIPVSLANCTKLVKLNLRVNQLGGTLSAIDFSRFQSLSILDLGNNSFTGEFPSTVYSCKMMTAMRFAGNKLTGQISPQVLELESLSFFTFSDNKMTNLTGALSILQGCKKLSTLIMAKNFYDETVPSNKDFLRSDGFPSLQIFGIGACRLTGEIPAWLIKLQRVEVMDLSMNRFVGTIPGWLGTLPDLFYLDLSDNFLTGELPKELFQLRALMSQKAYDATERNYLELPVFVNPNNVTTNQQYNQLSSLPPTIYIKRNNLTGTIPVEVGQLKVLHILELLGNNFSGSIPDELSNLTNLERLDLSNNNLSGRIPWSLTGLHFLSYFNVANNTLSGPIPTGTQFDTFPKANFEGNPLLCGGVLLTSCDPTQHSTTKMGKGKVNRTLVLGLVLGLFFGVSLILVLLALLVLSKRRVNPGDSENAELEINSNGSYSEVPPGSDKDISLVLLFGNSRYEVKDLTIFELLKATDNFSQANIIGCGGFGLVYKATLDNGTKLAVKKLTGDYGMMEKEFKAEVEVLSRAKHENLVALQGYCVHDSARILIYSFMENGSLDYWLHENPEGPAQLDWPKRLNIMRGASSGLAYMHQICEPHIVHRDIKSSNILLDGNFKAYVADFGLSRLILPYRTHVTTELVGTLGYIPPEYGQAWVATLRGDVYSFGVVMLELLTGKRPMEVFRPKMSRELVAWVHTMKRDGKPEEVFDTLLRESGNEEAMLRVLDIACMCVNQNPMKRPNIQQVVDWLKNIEAEKNQNNREEPEEEEET

>Brassica_ rapa-PSY1R- XP_009105890

MIDEKKMRSTKSICLLVRPVPMFLFLLIYVLSISVFFLTVSEAVCNLQDRDSLLFFSSNVSSPASPLHWSSSTDCCSWEGISCDDSPQNRVTSILLPSRGLSGNLPSSVLDLPRLTRLDLSHNRLSGPLPQGFFSVLDHLTFLDLSYNSFNGELPLEANGTSRNFPIQTVDLSSNFLQGQILSGSVFLQGAFNLTSFNVSNNSFTGPLPSFMCTTSPQLTKLDFSYNKFSGDISGGLGRCLKLNSLRAGFNNLSGEIPKEVYNLSELEEFSLPVNHLSGRIDDGITRLTKLTLLELYFNQLQGDIPKDIGRLINLRSLRLHINNLTGFVPVSLSNCTKLEKLNLRVNRLGGTLSVDFSRFQSLSILDLGNNSFTGDFPSTVYSCRNMTAMRFAGNKLTGQISPQVLELKSLTFFTFSDNNMTNITGALSILQGCKNLSTLIIAKNFYDETIPSNEDFLASGAFPKLQIFGTGGSRLKGEIPAWLIKLKSVELMDLSQNRFVGSIPGWLGTLPNLFYLDLSDNLLTGELPKELFQLRALMSQKVYDATERTYLELPVFVKPNNITSNQQYNQLASLPPAIYIRRNNLTGSIPVEIGQLKVLMHLELLGNKFSGSIPDELSNLTSLERLDLSNNNLSGRIPWSLTGLHFMSYFNVANNTLSGQIPTGSQFDTFPKSYFEGNPLLCGRVLQLSCTVAPKPYTNEKASTTVVLGIVIGIFFGVSLILVMLALWVMSKRRVNPGDSENAELEINSNASYSEVPPGSEKDISLVLLFGNSRYEVKDLTIFELLKATNNFSQANIIGCGGFGLVYKAVLDNGTKLAVKKLTGDYGLMEKEFKAEVEVLSRAKHENLVALQGYCVHDSARILIYSFMENGSLDYWLHENPEGPAQLDWAKRLHIMRGASCGLAYMHQICEPHIVHRDIKSSNILLDGSFKAYLADFGLSRLILPYRTHVTTELVGTLGYIPPEYGQAWVATLRGDVYSFGVVMLELLTGKRPMEVFRPKMSREIVAWVNQMRREEKPEEVFDPLLRESGHEREMLRVLDIACMCVNQNPMKRPVIQQVVDWLNDVDAGNRNQSNREEAEEEEETK

>Glycine_ max-PSY1R-XP_003520891

MTQGTRPFSIFMVSKLMVFVLILFLLSGFLVLVQASSCNQLDRDSLLSFSRNISSPSPLNWSASSVDCCSWEGIVCDEDLRVIHLLLPSRALSGFLSPSLTNLTALSRLNLSHNRLSGNLPNHFFSLLNHLQILDLSFNLFSGELPPFVANISGNTIQELDMSSNLFHGTLPPSLLQHLADAGAGGSLTSFNVSNNSFTGHIPTSLCSNHSSSSSLRFLDYSSNDFIGTIQPGLGACSNLERFRAGSNSLSGPLPGDIFNAVALTEISLPLNKLNGTIGEGIVNLANLTVLELYSNNFTGPIPSDIGKLSKLERLLLHANNITGTLPTSLMDCANLVMLDVRLNLLEGDLSALNFSGLLRLTALDLGNNSFTGILPPTLYACKSLKAVRLASNHFEGQISPDILGLQSLAFLSISTNHLSNVTGALKLLMELKNLSTLMLSQNFFNEMMPDDANITNPDGFQKIQVLALGGCNFTGQIPRWLVNLKKLEVLDLSYNQISGSIPPWLNTLPELFYIDLSFNRLTGIFPTELTRLPALTSQQAYDEVERTYLELPLFANANNVSQMQYNQISNLPPAIYLGNNSLNGSIPIEIGKLKVLHQLDLSNNKFSGNIPAEISNLINLEKLYLSGNQLSGEIPVSLKSLHFLSAFSVAYNNLQGPIPTGGQFDTFSSSSFEGNLQLCGSVVQRSCLPQQGTTARGHRSNKKLIIGFSIAACFGTVSFISVLIVWIISKRRINPGGDTDKVELESISVSSYSGVHPEVDKEASLVVLFPNKTNEIKDLTIFEILKATENFSQANIIGCGGFGLVYKATLPNGTTVAIKKLSGDLGLMEREFKAEVEALSTAQHENLVALQGYCVHEGVRLLIYTYMENGSLDYWLHEKADGPSQLDWPTRLKIAQGASCGLAYMHQICEPHIVHRDIKSSNILLDEKFEAHVADFGLARLILPYQTHVTTELVGTLGYIPPEYGQAWVATLRGDVYSFGVVMLELLSGRRPVDVSKPKMSRELVAWVQQMRSEGKQDQVFDPLLRGKGFEEEMQQVLDAACMCVNQNPFKRPSIREVVEWLKNVGSSKPQMNKD

>Nicotiana_sylvestris-PSY1R-XP_009759322

MITDNKDSRHLQSGLTYHQFRAAMLLTSLHSSSSSSSSSSLPHRNSFYLITVTVLVLLISSVATICHASCNQLDRDSLLSFSVAISSPSPLNWSSSFDCCTWEGVGCDNSGRVISLLLPSRSLFGSIRPSIANLSKLEQLSLSHNRFFGPLPDGFFESFSSLQIIDLSYNRLSGQLPLSDRLPSPIQLLNLSSNHFNGTIRSSFLEPAINLVSFDISNNSFSGQIPSFICSYSAAIRVLDFSSNEFVGQIPKGFGSCSNLVTLRAGFNHLSGSIPDDIYSVSTLQEIFLPANKFSGPMPEGIVNLVNLRILALYGNELTGLIPQDIGRLTKLEQLLLHINFLNGTVPPSLMACTRLTVLNLRVNFLEGELSALDFSNLSRLGTIDLGNNLFTGSIPQSLFSCRSLTAIRLATNKLTGDIMPGIMSLQSLSFLSVSNNSLTNFAGAIEVLKGCKNLTTLILTINFYNETLPDDGNLIGSEDFQNLQILGLGGCNFTGQIPTWLVKLRKLEVLDLSMNQITGKIPGWLGTLQNLFYMDLSQNLLYGGFPIELTQLQRLASEGAADQIERSALELPVFVQPNNASNQQYNQLSNLPPAIYLGHNSLDSIIPTEIGQLKYILVLDLSNNNFSGNIPETISNLTNLEKLDLSGNNLSGEIPSSLKGLHFLSSFSVAHNNLEGPIPTGGQFDTFPVTSFLGNPGLCGQILQHSCTDQSATTQPSAVRKSPKMKIIIGLILGISFGIALTLIVTALWIFSKRRILPRGDAEKNDLDIVSYNSTSGLSAENGKDNSMLVMFPTNKNQIKDLTIFDILKATNNFNQANIIGCGGFGLVYKATLADGTTLAVKKLSGDMGLIEREFRAEVEALSTAQHENLVSLEGYCVHDGCRLLFYSYMENGSLDYWLHEKTDGASLLDWPTRLKIAQGASFGLAYMHQICEPHIVHRDIKSSNILLDEKFKAHVADFGLSRLILPYHTHVTTELVGTLGYIPPEYSQSWIATLRGDVYSFGVVMLELLAGRRPVDMTKSKMSRELVVWVQQMRNEGKQEEIFDPLLRDKGFEEEMLKVLDVACMCVNHNPFKRPAITEVVEWLRGVGSKREAPK

>Sorghum_bicolor-PSY1R-XP_002437497

MQSLNLRCSSSSTSKLSVPFFGTALVLLLSYASLASSCTEQEKSSLIDFRDGLSQEGNGGLNMSWANSTDCCQWEGINCGNGGVVTEVLLPSKGLKGRIPPSLSNLTGLLHLNLSCNSLYGSLPAELVFSSSIIILDVSFNSLSGPLLERQSPISGLPLKVLNISSNSFTGQLPSTTLQVMNNLVALNASNNSFTGPLPSSICIHAPSLVILDLFLNDFSGTISPEFGNCSKLTVLKAGRNNLTGGLPHELFNATSLEHLAFPNNNLQGPLDGSSLVKLSNLIFLDLGSNGLEGEMPNSIGQLGRLEELHLDNNLMIGELPSALSNCRSLKYITLRNNSFMGDLSRVNFTQMDLRTADFSVNKFNGTIPESIYACSNLVALRLAYNNFHGQFSPRIANLRSLSFLSVTNNSFTNITDALQNLNRCKNLTSLLIGTNFKGETIPQDAAFDGFENLRVLTIDACPLVGEIPLWLSQLTKLEILDLSYNHLTGTIPSWINSLELLFFLDISSNRLTGDIPPELMEMPMLQSDKNTAKLDPKFLELPVFWTQSRQYRLLNAFPNVLNLCNNSLTGIIPQGIGQLKVLNVLNFSSNSLSGEIPQQICNLTNLQTLDLSNNQLTGELPTALSNLHFLSWFNVSNNDLEGPVPSGGQFNTFTNSSYIGNSKLCGPMLSVHCDPVEGPTTPMKKRHKKTIFALALGVFFGGLAMLFLLGRLILFIRSTKSADRNKSSNNRDIEATSFNSVSEHLRDMIKGSILVMVPRGKGESNNITFNDILKATNNFDQQNIIGCGGNGLVYKAELPCGSKLAIKKLNGEMCLMEREFKAEVEALSMAQHENLVPLWGYCIQGNTRLLIYSFMENGSLDDWLHNKDNANSFLDWPTRLKIAQGAGRGLSYIHNTCNPNIVHRDVKSSNILLDREFNAYVADFGLARLILPYNTHVTTELVGTLGYIPPEYGQAWVATLRGDIYSFGVVLLELLTGKRPVQVLTKSKELVQWVKEMRSQGKDIEVLDPALRGRGHDDQMLNVLEVACKCINHNPGLRPTIQEVVYCLETVVEPLQVQVQVQL

> Setaria_ italica-PSY1R-XP_004966176

MRPLNLPCCSSSSSKLPVPSFGLAFVLLLSTASFVSSCTEQERSSLIDFRDGLSLEGNGGLNNSWINGTDCCQWDGITCTNSVVTEIMLASKGLQGKISPSLGNLTGLLHLNLSRNSLYGSLPANLLFSSSIIILDVSFNHLSGPLLEQRSSNPGLPLQVLNISSNFFTGQLPSTTLEVMKNLVALNASNNSLMGPMPSSICNNAPSLAMLDICLNEFSGTISSEFGNCSMLKVLKAGHNNLTGVLPHELFNATSLEQLSFPNNDLQGILDASNLVKLANLIILDLGSNGLRGNIPDSIGQLRRLEELHLDNNLMSGELPLALGNCTRLKYITLRNNSFRGDLSTVNFAQLDLRIADFSINKFTGTIPESIYACSNLIALRLAYNNFIGQFSPRIGNLRSLSFLSITNNSFTNITDALQKLKSCKNLTSLLIGTNFKGETIPQDEAIDGFENLQVLTIDACPLVGKIPVWLSKLTKLEILDLSINQLTGSIPSWINGLKFLFFLDISSNKLTGDIPTTLMEMPMLQSEKNAAKLDPKLLELPVYWTQSRQYRVLNAFPSVLNLCNNRFTGIIPREIGHLKMLDVLNFSTNSFSGEIPQEICNLTNLQTLDLSNNQFTGPIPSALSNLHFLSWFNVSNNELEGPVPTGGQFNTFTNSSYSGNSKLCGSMLSTHCNSVQAPPASMRRKHNKGIVALALCVFFGGLAILFLLGRLILSIRRTKSADRNKGSNSRDIEATSFNSVSDHLCDGIKGSILVMVPRGKGESNKLTFSDILNATNNFDQQNIIGCGGNGLVYRAELPCGSKLAIKKLNGEMCLMEREFKAEVEALSMAQHENLVPLWGYCIQGSSRLLIYSFMENGSLDDWLHNKDDPNSFLDWPIRLKIAQGAGRGLSYIHNTCKPHIIHRDVKSSNILLDREFNAYVADFGLARLILPYDTHVTTELVGTLGYIPPEYGQAWVATLRGDIYSFGVVLLELLTGKRPVQVLTKSKELVQWVREIRSQGKDVEVLDPALRGRGHDDQMLNVLEVACKCINHNPCLRPTIQEVVSCLDSVDVNLQVQT

>Oryza_sativa_ Indica Group-PSY1R-AAU12600

MQPPHSSYKTQSNRLPIPVLSLALVLLLNFTSPTSSCTEQEKNSLLNFLTGLSKDGGLSMSWKDGVDCCEWEGITCRPDRTVTDVSLASRRLEGHISPYLGNLTGLLQLNLSHNQLSGALPAELVFSSSLIIIDVSFNRLNGGLNELPSSTPARPLQVLNISSNLLAGQFPSSTWEVMKNLVALNASNNSFTGQIPTNLCTNSPSLAVLELSYNQLSGSIPSELGNCSMLRVLKAGHNNLSGTLPNELFNATSLECLSFPNNGLEGNIDSTSVVKLSNVVVLDLGGNNFSGMIPDSIGQLSRLQELHLDHNNMHGELPSALGNCKYLTTIDLRGNSFSGDLGKFNFSTLLNLKTLDIGINNFSGKVPESIYSCSNLIALRLSYNNFHGELSSEIGKLKYLSFLSLSNNSFTNITRALQILKSSTNLTTLLIEHNFLEEVIPQDETIDGFKNLQVLTVGQCSLSGRIPLWLSKLTNIELLDLSNNQLTGPIPDWIDSLNHLFFLDISNNSLTGEIPITLMGMPMIRTAQNKTYLDPSFFELPVYVDKSLQYRILTAFPTVLNLSQNNFMGVIPPQIGQLKMLVVLDFSYNNLSGKIPESICSLTSLQVLDLSNNHLTGSIPGELNSLNFLSAFNVSNNDLEGPIPTGAQFNTFPNSSFDGNPKLCGSMLIHKCKSAEESSGSKKQLNKKVVVAIVFGVFLGGTVIVLLLGHFLSSLRAAIPKTENKSNSSGDLEASSFNSDPVHLLVMIPQGNTEANKLTFTDLVEATNNFHKENIIGCGGYGLVYKAELPSGSKLAIKKLNGEMCLMEREFAAEVEALSMAQHANLVPLWGYCIQGNSRLLIYSYMENGSLDDWLHNREDETSSFLDWPTRFKIARGASQGLLYIHDVCKPHIVHRDIKSSNILLDKEFKAYVADFGLSRLILPNKNHVTTELVGTLGYIPPEYGQAWVATLRGDVYSFGVVLLELLTGRRPVSILSTSKELVPWVLEMRSKGNLLEVLDPTLHGTGYEEQMLKVLEVACKCVNCNPCMRPTIREVVSCLDSIGSD

>Zea_mays-BIR1-NP_001147794

MTDHFALGLGALLLLLLSSSCFSSDLDVQCLRDVKQSVTDPTGILKSSWVFDNTSVGFICKFPGVECWYPDENRVLALRLSNFGLQGPFPKGLKNCTSMTTLDLSSNSFTGAIPSDIQQQVPFLASLDLSYNGFSGGIPVLIYNITYLNTLNLQHNQLSGDIPGQFSALARLQEFNVADNQLSGTIPSSLQKFPASNFAGNDGLCGPPLGECQASAKSKSTASIIGAVVGVVVVVIIGAIVVFFCLRRVPAKKAAKDEDDNNWAKSIKGTKTIKVSMFENPVSKMKLSDLMKATDEFSKENIIGTGRTGTMYRAVLPDGSFLAVKRLQDSQHSESQFASEMKTLGQVRHRNLVPLLGFCVAKKERLLVYKHMPLGSLYDQLNKEEGSKMDWALRLRIGIGAAKGLAYLHHTCNPRVLHRNISSKCILLDEDYEPKISDFGLARLMNPIDTHLSTFVNGEFGDLGYVAPEYARTLVATPKGDVYSFGVVLLELVTGERPTHVSSAPENFRGSLVEWISHLSNNALLQDAIDKSLVAKDADGELMQFLKVACSCTLATPKERPTMFEVYQLLRAIGERYHFTADDDLVLPPLSTDSDGVTLDELIVAK

>Arabidopsis_ thaliana-BIR1-NP_568696

MMMGRLVFVIWLYNCLCLLLLSSLVDADQANIDCLRTFKSQVEDPNRYLSTWVFGNETAGYICKFSGVTCWHDDENRVLSIKLSGYGLRGVFPPAVKLCADLTGLDLSRNNFSGPLPANISTLIPLVTILDLSYNSFSGEIPMLISNITFLNTLMLQHNQFTGTLPPQLAQLGRLKTFSVSDNRLVGPIPNFNQTLQFKQELFANNLDLCGKPLDDCKSASSSRGKVVIIAAVGGLTAAALVVGVVLFFYFRKLGAVRKKQDDPEGNRWAKSLKGQKGVKVFMFKKSVSKMKLSDLMKATEEFKKDNIIATGRTGTMYKGRLEDGSLLMIKRLQDSQRSEKEFDAEMKTLGSVKNRNLVPLLGYCVANKERLLMYEYMANGYLYDQLHPADEESFKPLDWPSRLKIAIGTAKGLAWLHHSCNPRIIHRNISSKCILLTAEFEPKISDFGLARLMNPIDTHLSTFVNGEFGDFGYVAPEYSRTMVATPKGDVYSFGVVLLELVTGQKATSVTKVSEEKAEEENFKGNLVEWITKLSSESKLQEAIDRSLLGNGVDDEIFKVLKVACNCVLPEIAKQRPTMFEVYQLLRAIGESYNFTADDDILIPSESGEGDFIEELIVAR

>Sorghum_ bicolor-BIR1-XP_002450551

MAVWCSCSAVLPVLFCFMICQLCYGTVTDIQCLKKLKASVDPDNKLEWTFNNNTEGSICGFNGVECWHPNENRVLSLHLGSFGLKGQFPDGLENCSSMTSLDLSSNNLSGPIPADISKRLPFITNLDLSYNSFSGEIPEALANCSYLNIVSLQHNKLTGTIPGQLAALNRLAQFNVADNQLSGQIPSSLSKFPASNFANQDLCGRPLSNDCTANSSSRTGVIVGSAVGGAVITLIIVAVILFIVLRKMPAKKKLKDVEENKWAKTIKGAKGAKVSMFEKSVSKMKLNDLMKATDDFTKDNIIGTGRSGTMYRATLPDGSFLAIKRLQDTQHSEDQFTSEMSTLGSVRQRNLVPLLGYCIAKNERLLVYKYMPKGSLYDNLHQQNSDKKALEWPLRLKIAIGSARGLAWLHHSCNPRILHRNISSKCILLDDDYEPKISDFGLARLMNPIDTHLSTFVNGEFGDLGYVAPEYTRTLVATPKGDVYSFGVVLLELVTREEPTHVSNAPENFKGSLVDWITYLSNNSILQDAIDKSLIGKGNDAELLQCMKVACSCVLSSPKERPTMFEVYQLLRAVGEKYHFSAADDELTMQPQNANPEDELIVAN

>Zea_ mays-OsSERK- XP_008678722

MAAAGRWWAVVLAVAVLLWPGRVVANTEGDALYSLRQSLIDTNNVLQSWDSTLVNPCTWFHVTCNSDNSVIRVDLGNAQLSGVLVPQLGQLKNLQYLELYSNKISGAIPPELGNLTNLVSLDLYMNNFSGNIPDRLGNLLKLRFLRLNNNSLVGPIPVALTNISTLQVLDLSSNNLSGPVSSNGSFSLFTPISFNNNPNLCGPVTTKPCPGDPPFSPPPPFNPPSPPTQSTGASGPGAIAGGVAAGAALVFAVPAIAFAMWRRRKPEEHFFDVPAEEDPEVHLGQLKKFSLRELQVATDTFSNKHILGRGGFGKVYKGRLADGSLVAVKRLKEERTPGGELQFQTEVEMISMAVHRNLLRLRGFCMTPTERLLVYPYMANGSVASRLRERQASEPPLKWETRRRIALGSARGLSYLHDHCDPKIIHRDVKAANILLDEEFEAVVGDFGLAKLMDYKDTHVTTAVRGTIGHIAPEYLSTGKSSEKTDVFGYGIMLLELITGQRAFDLARLANDDDVMLLDWVKGLLKEKKVEMLVDPDLQNAYEEIEVENLIQVALLCTQGSPLERPKMSEVVRMLEGDGLAERWDEWQKVEVVRQEAESAPLRNDWIVDSTYNLRAVELSGPR

>Oryza_ sativa_ Japonica Group-OsSERK-AAU88198

MAEARLLRRRRLCLAVAVVWVVAVAVSRVGANTEGDALYSLRQSLKDANNVLQSWDPTLVNPCTWFHVTCNPDNSVIRVDLGNAQLSGALVPQLGQLKNLQYLELYSNNISGTIPNELGNLTNLVSLDLYLNNFTGFIPETLGQLYKLRFLRLNNNSLSGSIPKSLTNITTLQVLDLSNNNLSGEVPSTGSFSLFTPISFANNKDLCGPGTTKPCPGAPPFSPPPPFNPPTPTVSQGDSKTGAIAGGVAAAAALLFAVPAIGFAWWRRRKPEEHFFDVPAEEDPEVHLGQLKRFSLRELQVATDNFSNKNILGRGGFGKVYKGRLADGSLVAVKRLKEERTPGGELQFQTEVEMISMAVHRNLLRLRGFCMTPTERLLVYPYMANGSVASRLRERQPNDPPLEWQTRTRIALGSARGLSYLHDHCDPKIIHRDVKAANILLDEDFEAVVGDFGLAKLMDYKDTHVTTAVRGTIGHIAPEYLSTGKSSEKTDVFGYGIMLLELITGQRAFDLARLANDDDVMLLDWVKGLLKEKKVEMLVDPDLQSGFVEHEVESLIQVALLCTQGSPMDRPKMSEVVRMLEGDGLAERWEEWQKVEVVRQEAELAPRHNDWIVDSTYNLRAMELSGPR

>Vitis_ vinifera-OsSERK-XP_002270847

MEGEVVVLFTLLLLCLLHPFSFISANMEGDALHTLRTNLEDPNNVLQSWDPTLVNPCTWFHVTCNSDNSVIRVDLGNAALSGQLVPQLGLLKNLQYLELYSNNISGPIPSDLGNLTSLVSLDLYLNSFTGPIPETLGKLSKLRFLRLNNNSLTGTIPMSLTNITALQVLDLSNNRLSGVVPDNGSFSLFTPISFANNLDLCGPVTGHPCPGSPPFSPPPPFVPPPPISSQGGNSATGAIAGGVAAGAALLFAAPAIGFAWWRRRKPQEYFFDVPAEEDPEVHLGQLKRFSLRELQVATDSFSNKNILGRGGFGKVYKGRLADGSLVAVKRLKEERTPGGELQFQTEVEMISMAVHRNLLRLRGFCMTPTERLLVYPYMANGSVASCLRERPASEPPLDWPTRKRIALGSARGLSYLHDHCDPKIIHRDVKAANILLDEEFEAVVGDFGLAKLMDYKDTHVTTAVRGTIGHIAPEYLSTGKSSEKTDVFGYGIMLLELITGQRAFDLARLANDDDVMLLDWVKGLLKEKKLEMLVDPDLKNNYVEAEVEQLIQVALLCTQGSPMDRPKMSEVVRMLEGDGLAERWDEWQKVEVLRQEVELAPHSNSDWIVDSTDNLHAVELSGPR

>Solanum_tuberosum-OsSERK-NP_001275293

MVKVMEKDAVVVSLVVWLILVVHHLKLIYANMEGDALHSLRVNLQDPNNVLQSWDPTLVNPCTWFHVTCNNDNSVIRVDLGNAALSGLLVPQLGLLKNLQYLELYSNNISGLIPSDLGNLTNLVSLDLYLNNFVGPIPDSLGKLSKLRFLRLNNNSLTGNIPMSLTNISSLQVLDLSNNRLSGVVPDNGSFSLFTPISFANNLDLCGPVTGRPCPGSPPFSPPPPFVPPPPISAPGGNGATGAIAGGVAAGAALLFAAPAIAFAWWRRRKPQEYFFDVPAEEDPEVHLGQLKRFSLRELQVATDSFSNKNILGRGGFGKVYKGRLADGSLVAVKRLKEERTPGGELQFQTEVEMISMAVHRNLLRLRGFCMTPTERLLVYPYMANGSVASCLRERPPSEPPLXWPXRKRIALGSARGLSYLHDHCDPKIIHRDVKAANILLDEEFEAVVGDFGLAKLMDYKDTHVTTAVRGTIGHIAPEYLSTGKSSEKTDVFGYGIMLLELITGQRAFDLARLANDDDVMLLDWVKGLLKEKKLEMLVDPDLQNKYVEAEVEQLIQVALLCTQSNPMDRPKMSEVVRMLEGDGLAERWDEWQKVEVLRQEVELAPHPGSDWLVDSTENLHAVELSGPR
